# *Francisella novicida* Pathogenicity Island Encoded Proteins Were Secreted during Infection of Macrophage-Like Cells

**DOI:** 10.1371/journal.pone.0105773

**Published:** 2014-08-26

**Authors:** Rebekah F. Hare, Karsten Hueffer

**Affiliations:** 1 Department of Biology and Wildlife, Institute of Arctic Biology, University of Alaska Fairbanks, Fairbanks, Alaska, United States of America; 2 Department of Veterinary Medicine, University of Alaska Fairbanks, Fairbanks, Alaska, United States of America; University of Louisville, United States of America

## Abstract

Intracellular pathogens and other organisms have evolved mechanisms to exploit host cells for their life cycles. Virulence genes of some intracellular bacteria responsible for these mechanisms are located in pathogenicity islands, such as secretion systems that secrete effector proteins. The *Francisella* pathogenicity island is required for phagosomal escape, intracellular replication, evasion of host immune responses, virulence, and encodes a type 6 secretion system. We hypothesize that some *Francisella novicida* pathogenicity island proteins are secreted during infection of host cells. To test this hypothesis, expression plasmids for all *Francisella novicida* FPI-encoded proteins with C-terminal and N-terminal epitope FLAG tags were developed. These plasmids expressed their respective epitope FLAG-tagged proteins at their predicted molecular weights. J774 murine macrophage-like cells were infected with *Francisella novicida* containing these plasmids. The FPI proteins expressed from these plasmids successfully restored the intramacrophage growth phenotype in mutants of the respective genes that were deficient for intramacrophage growth. Using these expression plasmids, the localization of the *Francisella* pathogenicity island proteins were examined via immuno-fluorescence microscopy within infected macrophage-like cells. Several *Francisella* pathogenicity island encoded proteins (IglABCDEFGHIJ, PdpACE, DotU and VgrG) were detected extracellularly and they were co-localized with the bacteria, while PdpBD and Anmk were not detected and thus remained inside bacteria. Proteins that were co-localized with bacteria had different patterns of localization. The localization of IglC was dependent on the type 6 secretion system. This suggests that some *Francisella* pathogenicity island proteins were secreted while others remain within the bacterium during infection of host cells as structural components of the secretion system and were necessary for secretion.

## Introduction

Pathogenicity islands exist in many pathogenic bacteria, are acquired via horizontal gene transfer, and encode genes that facilitate interactions with host cells [1]. Secretion systems in bacteria involve the transport or translocation of effector molecules from the interior of a bacterial cell through its membranes to the exterior. Protein secretion is an important mechanism for bacteria to adapt and survive in their environment, including within an infected host [2]. Effector proteins are enzymes or toxins that facilitate infection and are secreted by these secretion systems [3].

*Francisella tularensis* is an intracellular pathogen that possesses the *Francisella* pathogenicity island (FPI) [4]. The FPI is found in all *Francisella* species and strains, and is duplicated in all human-virulent biovars of *F. tularensis. F. novicida* and *F. philomiragia* harbor only one copy of the FPI, which makes these species attractive for creating isogenic FPI gene deletion mutants [4], [5]. The molecular mechanisms contributing to the intracellular survival of *Francisella* are poorly understood, and FPI mutagenesis approaches are useful in identifying genes required for intracellular replication and virulence [4], [6], [7], [8], [9], [10], [11].

The FPI contains genes with homology to genes encoding type 6 secretion systems (T6SS) in other bacteria [12], [13], [14], [15]. Bioinformatics, genetics, biochemical, and cell biology approaches provide evidence the FPI encodes a functional secretion system [12], [13]. Homologues of *iglAB, pdpB, dotU*, and *vgrG* are found in most T6SS identified to date; therefore, some suspect the secretion system of the FPI is a T6SS, although this is debatable [15], [16]. DotU and PdpB are inner membrane components that are homologous with the T6SS proteins DotU and IcmF, respectively [15]. IglA and IglB are IcmF-associated homologous proteins seen in *Rhizobium leguminosarum*, *Salmonella enterica*, and *Vibrio cholerae* [4], [12], [13], [15], [16], [17], [18]. The solubility properties of IglABC suggest these proteins could be part of the needle spanning through the bacterial membranes, and the protein-protein interactions of IglAB also suggests the auxiliary roles within *F. novicida* as described in other species [13]. Mutations in IglA and IglB result in bacteria that are unable to escape the phagosome and unable to replicate intracellularly [4], [6], [12], [19], [20]. In some species, these homologues are responsible for secretion of proteins, including Hcp and VgrG [16], [18], [21], [22], [23]. Recent studies suggest the T6SSs constitute important *Francisella* virulence, intracellular growth, or survival factors; however, only basic aspects of this system have been characterized [13], [24], [25].

Although the ability of *F. tularensis* to replicate within macrophages is multifactorial, our working hypothesis is that *F. tularensis* secretes FPI-encoded proteins that facilitate the organism's ability to escape the vacuole, enter the cytoplasm to replicate intracellularly, and down regulate the host immune cytokine response. If this is correct, then FPI-encoded proteins should be secreted during infection within host macrophages. Currently available genetic tools for studying the FPI-encoded proteins consist of green fluorescent protein (GFP) tags [8] and more recently reporter fusion tag systems [11]. Secretion of FPI-encoded proteins have previously been examined in the *Francisella* live vaccine strain (LVS) with a fusion β-lactamase, however, this system is not applicable to wild type *F. novicida* and was assessed in a β-lactamase gene mutant because *F. novicida* possesses native β-lactamase genes that exhibit the same activity toward the TEM substrate and interfere with the assay [11]. In the current study, FPI-encoded proteins were expressed as fusion proteins with the small triple FLAG tag and tracked within infected macrophage-like cells. The localization of IglC in a T6SS mutant was also assessed.

## Materials and Methods

### Bacterial and Cell Cultures

Bacterial strains and cell lines used in this study are listed in Table 1. *Escherichia coli* D10 (Invitrogen) was grown aerobically at 37°C on Luria-Bertani (LB) media, containing 50 µg/ml of ampicillin (LBA) when appropriate for selection and maintenance. *F. novicida* U112 (ATTC 15482) was cultured aerobically at 37°C on tryptic soy agar (TSA) or in tryptic soy broth (TSB) supplemented with 0.1% cysteine. When selecting for or maintaining transformants, *Francisella* was cultured on TSA containing 15 µg/ml of kanamycin. J774-1A murine macrophage-like cells were obtained from the American Type Culture Collection (ATCC, TIB 67, BALB/C macrophage). J774 cells were grown in flasks in Dulbecco's Modified Eagle Medium (DMEM) (GIBCO Invitrogen Grand Island, NY USA) supplemented with 10% newborn calf serum (NCS) and maintained at 37°C in a humidified 6.5% CO_2_ incubator. The mosquito hemocyte like cells Sua-1B were grown in Schneider's Insect Medium (Sigma Aldrich St. Louis, MO USA) supplemented with 20% fetal bovine serum at 28°C and flasks were capped tightly [26].

**Table 1:** List of Strains and Plasmids.

Strain	Description	Reference
J774-1A	Murine Macrophages cell lines	ATTC
Sua-1B	Mosquito hemocytes cell lines	[26]
*E. coli DH5α*	Sub cloning competent cells	Invitrogen
*F. novicida* U112	*Francisella novicida* prototype strain	ATTC
*F. novicida U112R 2008*	U112 Δ restriction genes	[40]
*Jlo*	U112 with deletion of gene FTN1758	[36]
Δ*pdpA*	U112 Δ*pdpA*	[32]
Δ*pdpB*	U112 Δ*pdpB*	[13]
Δ*iglE*	U112 Δ*iglE*	[13]
Δ*vgrG*	U112 Δ*vgrG*	[13]
Δ*iglF*	U112 Δ*iglF*	[13]
Δ*iglG*	U112 Δ*iglF*	[13]
Δ*iglH*	U112 Δ*iglH*	[13]
Δ*dotU*	U112 Δ*dotU*	[13]
Δ*iglI*	U112 Δ*iglI*	[13]
Δ*iglJ*	U112 Δ*iglJ*	[13]
Δ*pdpC*	U112 Δ*pdpC*	[13]
Δ*pdpE*	U112 Δ*pdpE*	[13]
Δ*iglD*	U112 Δ*iglD*	[13]
Δ*iglC*	U112 Δ*iglC*	[12]
Δ*iglB*	U112 Δ*iglB*	[13]
Δ*iglA*	U112 Δ*iglA*	[13]
Δ*pdpD*	U112 Δ*pdpD*	[36]

### DNA Manipulations

Restriction enzyme digests, sub-cloning, cloning, and DNA electrophoresis for *E. coli* was performed using standard cloning techniques [27] and the (Invitrogen Carlsbad, CA. USA) E-Gel Clonewell 0.8% SYBR Safe gel system. By cutting the *Francisella* expression plasmid groE-GFP- pFNLTP6 [8] with BamHI, the GFP insert was removed, leaving the groE promoter and the multiple cloning site (MCS) in place. Within the MCS a fragment containing *sopB* and a triple FLAG tag from the plasmid pSB2598 was inserted [28]. Next through quick-change mutagenesis, the second NcoI site in the plasmid's kanamycin resistance gene was removed without changing the coding sequence. By modifying this second NcoI site, most of the FPI genes have been inserted into plasmids using the restriction enzymes EcoRI and NcoI within the MCS; this allows for easy primer design and cloning of C-terminus triple FLAG plasmids. Primers used to construct the *Francisella* expression plasmids with the epitope tag on the C-terminus of FPI are listed in Table 2. After cloning all C-terminal tagged FPI genes, pKH8, IglA-FLAG, has been modified becoming the backbone for N-terminus tagged plasmids. These modifications involved removing *iglA*, leaving the triple FLAG tag down stream of the groE promoter and the Shine-Delgarno sequences of *iglA* yet upstream of the MCS. Primers were designed for N-terminus triple FLAG-tagged FPI genes to be inserted with the restriction enzymes Xma1 and Xho1 (Fig. S1). Primers used to construct the *Francisella* expression plasmids with the epitope tag on the N-terminus of FPI are listed in Table 3. PCR for cloning was done using Phusion High-Fidelity PCR (Finnzymes). Restriction enzyme digest was performed as described in New England Biolabs Catalog and Technical Reference (Ipswich, MA. USA). PCR products and restriction enzyme digest products were purified via Wizard SV Gel and PCR clean up System (Promega Madison, WI, USA). Ligations using T4 DNA ligase (Fisher Scientific) were done at 16°C for 14–16 h. Plasmids were recovered from *E. coli* through PureYield Plasmid Miniprep System (Promega Madison, WI, USA) for screening and PureYield Plasmid Midiprep System (Promega Madison, WI, USA) to collect a stock for transformations. Plasmids generated in this study are listed in Table 4. Plasmids were initially screened using restriction enzymes that were used for cloning and when applicable another restriction enzyme that would cut within the specific FPI gene. Plasmids were also screened for correct gene and triple FLAG sequence using standard Taq PCR. After plasmids and gene inserts were ligated, they were transformed into *E. coli* D10 using electroporation. Plasmids collected from *E. coli* were sequenced to confirm that the FPI gene sequence was not altered before being transformed into *F. novicida*.

**Table 2:** C-term Primers Used in this Study.

C-terminal FLAG *Francisella* expression plasmid primers
pdpA_C_terminalFLAG_F_Nde1: ggcagCATATGctaattaagtagacaatgatagc
pdpA_C_terminalFLAG_B_Nco1: ggcagCCATGGGatttccttttgatttatat
pdpB_C_terminalFLAG_F_KpnI1: agGGTACCcaaaaggaaattaaaagtatg
pdpB_C_terminalFLAG_B_Nco1: agCCATGGGttgtacattaacttctccttg
iglE_C_terminal_F_EcoR1: aggaGAATCCggcaaaaacaaggagaagttaatg
iglE_C_terminal_B_Nco1: gacgCCATGGCatctttttctatgctgctatc
vgrG_C_terminal_F_EcoR1: gagaGAATTCgattaaggggatattcttatg
vgrG_C_terminal_B_Nco1: agagCCATGGCtccaaccattgttgctgcggaacc
iglF_C_terminal_F_Nde1: gcagCATATGcaatggttggataataatatg
iglF_C_terminal_B_Nco1: agcaCCATGGCattttccaataagcttcttgcttgc
iglG_C_terminal_F_EcoR1: agagGAATTCgaagcttattggaaaatttaaatg
iglG_C_terminal_B_Nco1: agagCCATGGCagatgtttttacatttatttg
iglH_C_terminal_F_EcoR1: agagGAATTCcttagaaggtcattatcatg
iglH_C_terminal_B_Nco1: agagCCATGGCtatagagttatttaaaacaatc
dotU_C_terminal_F_EcoR1: aggaGAATTCctatataaaggatattagaaatg
dotU_C_terminal_B_Nco1: aggaCCATGGCccagcttaataaaattag
iglI_C_terminal_F_EcoR1: cgagGAATTCgggtaagaggagatttatatg
iglI_C_terminal_B_Nco1: gaagCCATGGCtatgtcaaaaagatcttc
iglJ_C_terminal_F_EcoR1: caagGAATTCcaaatgagatagatg
iglJ_C_terminal_B_Nco1: agcgCCATGGCtaaattaaaataacc
pdpC_C_terminal_F_EcoR1: ggcgaGAATTCgataaattaaggaagtacatatg
pdpC_C_terminal_B_Nco1: ggcagCCATGGGtgacgatatttttttaaaaaagtc
pdpE_C_terminal_F_EcoR1: gaagGAATTCcttaaggatgcaaaaatatg
pdpE_C_terminal_B_Nco1: aggcCCATGGCtattatagtaattttcttttc
iglD_C_terminal_F_EcoR1: ggcagGAATTCaagatcggagttgattctaatg
iglD_C_terminal_B_Nco1: ggcgaCCATGGGagaaaaggctataaagaaatc
iglC_C_terminal_F_EcoR1: gaGAATTCaaaggagaatgattatgagtgag
iglC_C_terminal_B_Nco1: gaCCATGGGtgcagctgcaatatatcc
iglB_C_terminal_F_Nde1: gagaCATATGgtagagaggattttgttatg
iglB_C_terminal_B_Nco1: agagCCATGGCgttattatttgtacc
iglA_C_terminal_F_EcoR1: agagGAATTCgtaaaaaaaggacaataagatg
iglA_C_terminal_B_Nco1: ggaaCCATGGCcttatcatctacttg
pdpD_C_terminal_F_EcoR1: ggcgaGAATTCgtaagagtagtaagtatggatcaag
pdpD_C_terminal_B_Nco1: ggcgaCCATGGGaacccagatcattggtctatac
anmK _C_terminal_F: agagGAATTCgaatataaatattgtgtaggaatcatg
anmK _C_terminal_B_Nco1: aggaCCATGGCaaagaaatttatttgacc

The description contains the FPI ORF nomenclature, which terminal the tag was fused to, direction of the primer, restriction enzyme used in cloning the respective ORF, the sequence of the primer in 5′-3′ direction, underlining represents in frame codon.

**Table 3:** N-term Primers Used in this Study.

N-terminal FLAG *Francisella* expression plasmid primers
pdpA_N_terminal_F_Xma1: acggCCCGGGgaatagcagtaaaagatataac
pdpA_N_terminal_B_Xho1: acggCTCGAGttaatttccttttgatttatatc
pdpB_N_terminal_F_Xma1: acggCCCGGGgaaattttattaaaaatcatc
pdpB_N_terminal_B_Xho1: acggCTCGAGttattgtacattaacttctccttg
iglE_N_terminal_F_Xma1: acggCCCGGGgatacaataaattattgaaaaatc
iglE_N_terminal_B_Xho1: acggCTCGAGttaatctttttctatgctgc
vgrG_N_terminal_F_Xma1: acggCCCGGGgatcaaaagcagaccatattttc
vgrG_N_terminal_B_Not1: caggGCGGCCGCttatccaaccattgttgctgcgg
iglF_N_terminal_F_Xma1: acggCCCGGGgaaataatgatattgataaatgg
iglF_N_terminal_B_Xho1: acggCTCGAGttaaattttccaataagcttcttgc
iglG_N_terminal_F_Xma1: acggCCCGGGgattaaatattataaatgactcc
iglG_N_terminal_B_Xho1: acggCTCGAGctaagatgtttttacatttatttgtcc
iglH_N_terminal_F_Xma1: acggCCCGGGatgaaaaaagaaaagatttaag
iglH_N_terminal_B_Xho1: acggCTCGAGttatatagagttatttaaaacaatc
dotU_N_terminal_F_Xma1: acggCCCGGGgaaaagactttaaagagatag
dotU_N_terminal_B_Xho1: acggCTCGAGttaccagcttaataaaattagtaagc
iglI_N_terminal_F_Xma1: acggCCCGGGgaagtcagataatatctacac
iglI_N_terminal_B_Xho1: acggCTCGAGttatatgtcaaaaagatcttc
iglJ_N_terminal_F_Xma1: acggCCCGGGgaaagactattttgaagatctttttg
iglJ_N_terminal_B_Xho1: acggCTCGAGtcataaattaaaataacctagatatatc
pdpC_N_terminal_F_Xma1: acggCCCGGGgaaacgacaaatatgaactaaatatc
pdpC_N_terminal_B_Xho1: acggCTCGAGctatgacgatatttttttaaaaaag
pdpE_N_terminal_F_Xma1: acggCCCGGGgaagtaaaaaagtatttcaattattattaatatttg
pdpE_N_terminal_B_Xho1: acggCTCGAGttatattatagtaattttcttttc
iglD_N_terminal_F_Xma1: acggCCCGGGgatttctagaaaggatttattg
iglD_N_terminal_B_Xho1: acggCTCGAGttaagaaaaggctataaagaaatc
iglC_N_terminal_F_Xma1: acggCCCGGGgaattatgagtgagatgataacaag
iglC_N_terminal_B_Xho1: acggCTCGAGctatgcagctgcaatatatcc
iglB_N_terminal_F_Xma1: acggCCCGGGgaacaataaataaattaag
iglB_N_terminal_B_Xho1: acggCTCGAGttagttattatttgtaccg
iglA_N_terminal_F_Xma1: acggCCCGGGcaaaaaataaaatcccaaattc
iglA_N_terminal_B_Not1: caggGCGGCCGCctacttatcatctacttgttgattac
pdpD_N_terminal_F_Xma1: acggCCCGGGatcaagatatcaacgatttattatatg
pdpD_N_terminal_B_Xho1: acggCTCGAGttaaacccagatcattggtctatac
anmk_N_terminal_F_Xma1: acggCCCGGGgatctggaacatcactagatgg
anmk_N_terminal_B_Xho1: acggCTCGAGttaaaagaaatttatttgacc

The description contains the FPI ORF nomenclature, which terminal the tag was fused to, direction of the primer, restriction enzyme used in cloning the respective ORF, the sequence of the primer in 5′-3′ direction, underlining represents in frame codon.

**Table 4:** Plasmids used in this study.

Plasmid	Description	Reference
pFNLTP6-gro-gfp	groE-gfp; Km^r^ Ap^r^	[8]
pKH1	Km^r^ Ap^r^	This study
pSB2598	sopB-FLAG	[28]
pKH2	Km^r^ Ap^r^	This study
pKH3	gro-sopB-FLAG; Km^r^ Ap^r^	This study
pKH22	gro-pdpA-FLAG; Km^r^ Ap^r^	This study
pKH24	gro-pdpB-FLAG; Km^r^ Ap^r^	[13]
pKH9	gro-iglE-FLAG; Km^r^ Ap^r^	[13]
pKH10	gro-vgrG-FLAG; Km^r^ Ap^r^	[13]
pKH26	gro-iglF-FLAG; Km^r^ Ap^r^	[13]
pKH11	gro-iglG-FLAG; Km^r^ Ap^r^	[13]
pKH12	gro-iglH-FLAG; Km^r^ Ap^r^	[13]
pKH13	gro-dotU-FLAG; Km^r^ Ap^r^	[13]
pKH14	gro-iglI-FLAG; Km^r^ Ap^r^	[13]
pKH15	gro-iglJ-FLAG; Km^r^ Ap^r^	[13]
pKH5	gro-pdpC-FLAG; Km^r^ Ap^r^	This study
pKH16	gro-pdpE-FLAG; Km^r^ Ap^r^	This study
pKH6	gro-iglD-FLAG; Km^r^ Ap^r^	This study
pKH4	gro-iglC-FLAG; Km^r^ Ap^r^	[13]
pKH18	gro-iglB-FLAG; Km^r^ Ap^r^	This study
pKH8	gro-iglA-FLAG; Km^r^ Ap^r^	This study
pKH7	gro-pdpD-FLAG; Km^r^ Ap^r^	This study
pKH25	gro-anmK-FLAG; Km^r^ Ap^r^	This study
pKH40	gro-FLAG-pdpA; Km^r^ Ap^r^	This study
pKH50	gro-FLAG-pdpB; Km^r^ Ap^r^	This study
pKH34	gro-FLAG-iglE; Km^r^ Ap^r^	This study
pKH35	gro-FLAG-vgrG; Km^r^ Ap^r^	This study
pKH44	gro-FLAG-iglF; Km^r^ Ap^r^	This study
pKH36	gro-FLAG-iglG; Km^r^ Ap^r^	This study
pKH45	gro-FLAG-iglH; Km^r^ Ap^r^	This study
pKH37	gro-FLAG-dotU; Km^r^ Ap^r^	This study
pKH39	gro-FLAG-iglI; Km^r^ Ap^r^	This study
pKH47	gro-FLAG-iglJ; Km^r^ Ap^r^	This study
pKH41	gro-FLAG-pdpC; Km^r^ Ap^r^	This study
pKH38	gro-LAG-pdpE; Km^r^ Ap^r^	This study
pKH48	gro-FLAG-iglD; Km^r^ Ap^r^	This study
pKH46	gro-FLAG-iglC; Km^r^ Ap^r^	This study
pKH43	gro-FLAG-iglB; Km^r^ Ap^r^	This study
pKH27	gro-FLAG-iglA; Km^r^ Ap^r^	This study
pKH42	gro-FLAG-pdpD; Km^r^ Ap^r^	This study
pKH49	gro-FLAG-anmK; Km^r^ Ap^r^	This study

This table lists all the plasmids used in designing the *Francisella* expression plasmids and all of the *Francisella* expression plasmids that were generated in this study.

### Transforming *Francisella*

These newly constructed *Francisella* expression plasmids containing the FPI encoded ORFs were chemically transformed into *F. novicida* strain U112. A sub-culture of bacteria was grown aerobically at 37°C, shaking at 200 rpm until mid log phase or an OD_600 nm_ of 0.3–0.5. Cells were pelleted at 5,000×*G* for 5 min at room temperature. Cells were suspended in *Francisella* transformation buffer or transformation medium [29] and then 400 µl of cell suspension was mixed with DNA and incubated aerobically at 37°C with shaking at 90 rpm for 1 h. 56 µl of 10% glucose and one ml of TSB was then added per transformation and incubated overnight aerobically at 37°C with shaking at 150 rpm. Cells were plated in 100 µl aliquots on freshly prepared TSA containing 15 µg/ml of kanamycin. Colonies were picked, isolated, and then screened by PCR, restriction enzyme digest, and Western blotting for confirmation of successful transformation.

### SDS-PAGE and Western Blotting

SDS-PAGE was performed using standard techniques [27]. Proteins were transferred to Immobilon-P membrane (Millipore Billerica, MA USA), and then blocked in 5% non-fat dry milk (NFDM) in Tris-Buffered Saline and Tween 20 (Fisher BioReagents Fair Lawn, NJ US) solution (TBST) containing 1 mM Tris, 15 mM NaCl, 2 mM KCl, and 0.1% Tween 20 for 1 h. To detect FLAG-tagged proteins, the blots were incubated with (1/5000) monoclonal M2 anti-FLAG antibodies (Sigma Aldrich St. Louis, MO USA) in 5% NFDM in TBST. For the detection of IglA, IglC, PdpA, and PdpC, polyclonal rabbit anti IglA, IglC, PdpA, and PdpC antibodies were used 1/2000. To detect bound antibodies, blots were incubated with Peroxidase-Goat Anti-Mouse or Peroxidase-Goat Anti-Rabbit secondary antibodies (1/5000) (Zymed Laboratories Invitrogen Immundetection San Francisco, CA USA) in 5% NFDM in TBST. To visualize protein bands, blots were incubated with SuperSignal West Pico Chemiluminescent Substrate (Thermo Scientific Rockford, IL USA) prior to exposing and developing film.

### Macrophage Growth Assay and Analysis

J774A.1 mouse macrophage like cells (ATCC TIB-67) were seeded in 24-well cell culture plates at 1.4×10^5^ cells/well for 24 h in complete Dulbecco's Modified Eagle Medium (cDMEM) containing 10% newborn calf serum (NCS). In 4 independent experiments, cells were infected in triplicate with *F. novicida* strains at a multiplicity of infection (MOI) of 1∶50 (bacterium-to-macrophage). To help promote bacterial uptake after bacteria have been added, the 24-well dishes containing infected macrophages were centrifuged at 600×*G* for 10 min. Infected monolayers were incubated for 2 h in DMEM to allow for phagocytosis to occur, washed five times in Hank's Phosphate Buffered Saline (HPBS) (GIBCO Invitrogen Grand Island, NY USA). At this time, the infection is at 0 h, and infected macrophages were then either lysed at this time or incubated at 37°C in 5% CO_2_ for 24 and 48 h. To determine bacterial replication, infected macrophages were lysed in 0.1% dexoxycholate in HPBS at 0, 24, and 48 h post infection. The lysates were serially diluted in HPBS and plated on TSA and incubated at 37°C for 24 or 48 h. The colony forming units (cfu) were enumerated, and used to plot growth curves. To perform statistical analysis, the fold replication at 48 h was first determined (cfu 48 h/cfu 0 h), and then the log of the 48 h fold replication was used in a two-way ANOVA with XLSTAT to compare the means of each group in Tukey multiple comparisons (α = 0.05).

### Immuno-fluorescence Microscopy and Analysis

J774 murine macrophage-like cells or Sua-1B mosquito hemocyte-like cells were grown on coverslips and infected with *F. novicida* strains as indicated in each figure (Table 1) [13]. Cells were infected for 30 min at an MOI of 50∶1 (bacteria per macrophage), washed with phosphate buffered saline (PBS), and incubated until the desired time point in DMEM containing 10% NCS. Cells were then fixed in 4% paraformaldehyde for 15 min at room temperature and rinsed three times with PBS. FLAG-tagged proteins and *Francisella* were detected with anti-FLAG M2 monoclonal antibodies and rabbit anti-*Francisella novicida* serum, respectively. The antibodies were diluted (1/500) in PBS containing 0.5% BSA and 0.1% saponin to permeate host cell membranes, while leaving the bacterial cell membranes intact [30]. Primary antibodies were detected with goat anti-mouse and goat anti-rabbit serum conjugated to Alexa Fluor 488 and 594, respectively (Invitrogen MOLECULAR PROBES Eugene, OR US). DNA was detected with DAPI (Invitrogen MOLECULAR PROBES Eugene, OR US). Coverslips were mounted using Prolong Gold Antifade reagent (Invitrogen MOLECULAR PROBES Eugene, OR US) and examined using an Olympus TE81 inverted fluorescent microscope with spinning disc confocal capabilities.

Images containing a total of 10–60 infected cells and 100–600 bacteria for each of the 3 independent experiments were collected as Z-stacks and a projection image was generated using the Intelligent Imaging SlideBook software package. Exposure time and settings were constant for all slides in each experiment. Using SlideBook software, masks were generated for infected macrophage-like cells, bacteria, and FLAG-tagged protein signals. The percentage of bacterial masks that overlapped with FLAG masks was used to determine the percentage of bacteria associated or co-localized with FLAG-tagged protein. However, this did not account for FLAG-tagged protein that dispersed away from bacteria, therefore the percentage of infected macrophage masks containing FLAG-tagged masks were also determined for every FPI protein with each the C-terminal and N-terminal FLAG-tag. Three independent experiments were performed. The data were analyzed in XLSTAT with an ANOVA paired with a left sided Dennett's test comparing each FLAG-tagged proteins' mean to the mean of bacteria not containing a FLAG expressing plasmid for either the percentage of bacteria co-localized with FLAG signal or the percentages of infected macrophage-like cells containing FLAG signal. Significant differences were determined with an α = 0.05. We also tested the data for correlations between the same proteins with different tags using the Spearman correlation test in XLSTAT. Significant differences were determined with an α = 0.05.

## Results

### FLAG-tagged FPI Protein Expression in *F. novicida*

**Figure 1:**
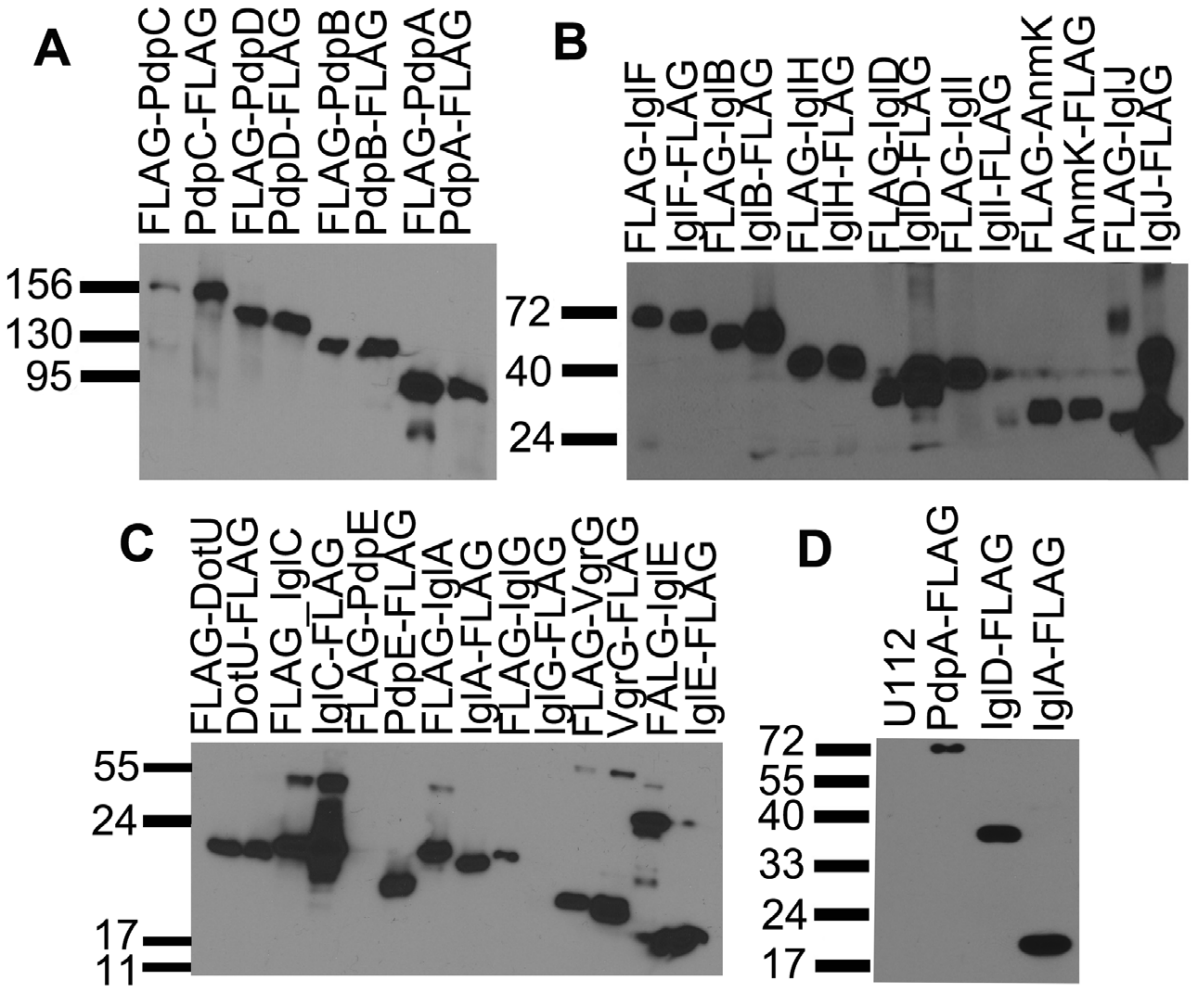
FPI FLAG-tagged Protein Expression in *F. novicida*. Whole cell lysates have been analyzed for production of N- terminal and C-terminal FLAG-tagged proteins by Western blotting. *F. novicida* U112 wild type and U112 expressing the respective FPI protein from the *Francisella* expression plasmid are labeled above each lane. C-terminally tagged proteins are referred to as protein-FLAG, and N-terminally tagged proteins are referred to as FLAG-protein. FPI encoded proteins have been grouped into similar predicted sizes (A) 156–95 kDa, (B) 67.6–30.9, (C) 24.6–14.5, and (D) a 10% gel for comparisons. FLAG-tagged proteins are detected with monoclonal mouse anti FLAG, goat anti mouse conjugated HRP, and chemiluminescent substrate.

Expression of C-terminal and N-terminal epitope tagged FPI proteins from *F. novicida* U112 was confirmed by Western blotting (Fig. 1). Western blotting showed the C-terminal and N-terminal tags do not interrupt FPI protein expression at their expected sizes (Fig. 1 and Table S1), with the exception of FLAG-PdpE. In addition to the expected sizes, lower intensity bands of different sizes were detected for some proteins (Fig. 1). IglG-FLAG expression was lower than the other FPI proteins and was not visible here (Fig. 1C); expression of IglG-FLAG was confirmed with longer exposure times causing over exposure with the other proteins (data not shown). Antibodies towards IglA, IglC, PdpA, and PdpC detected proteins of the same size as western blots detecting the FLAG-tag (Fig. S2).

### Intramacrophage Growth Complementation

**Figure 2:**
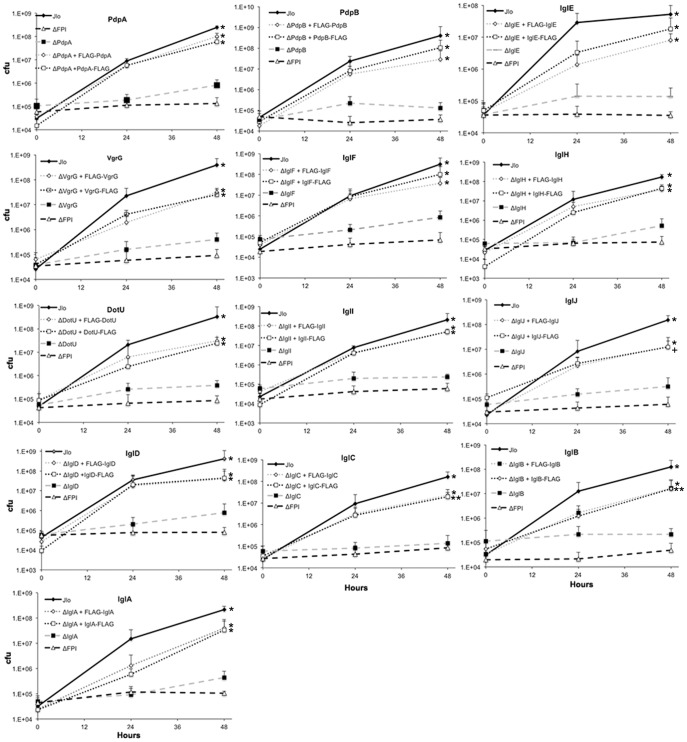
Intracellular Growth. J774 cells were infected with indicated strains and harvested at 0, 24 and 48 h post infection and the amount of intracellular bacteria at each time point was determined. The FPI mutant lacks all FPI genes and was defective for intracellular growth, as were each of the individual FPI gene mutants. All of the genetic complements express the cognate FPI gene as a C-terminal or an N-terminal triple FLAG-tagged version from a plasmid vector. Graphs are the average of four independent experiments and error bars show standard deviations, * indicate significance from mutants, ** partial complementation that was different form mutants and wild type, and + approaching significance at 0.054. P values generated by Tukey multiple comparisons via a two-way ANOVA.

Since several FPI genes are needed for intracellular growth, the C-FLAG and N-FLAG-tagged proteins ability to complement respective knock out mutant strains were assessed (Table 1). As previously described, the FPI deletion mutants of *iglABCDEFHIJ, pdpAB, dotU*, and *vgrG* were defective for intramacrophage growth [8], [12], [30], [32] (Fig. 2). Expression of C-terminal and N-terminal tagged FPI proteins, IglABCDEFHIJ, PdpAB, DotU, and VgrG, in FPI mutants increased growth rates, indicating that *Francisella* expression plasmids complemented their mutants. Genetic complementation of each deletion mutant with the C-FLAG and N-FLAG-tagged *Francisella* expression plasmids restored intramacrophage growth, and the growth of complemented mutants were significantly higher compared to their parental mutant (Fig. 2) (P<0.05). Expression of the tagged proteins did not always completely restore growth to that of the wild type; growth of 22 of the 26 complements were equivalent to that of the wild type (P>0.05). However, growth of the complements, FLAG-IglB, IglC-FLAG, IglJ-FLAG, and FLAG-IglJ, were significantly different compared to wild type and their respective mutant (P<0.05). Together, these data indicated that most of the plasmids expressed biologically functional proteins.

### Unique Patterns of FPI-Encoded Proteins Co-localized with *F. novicida* within Infected Cells

**Figure 3:**
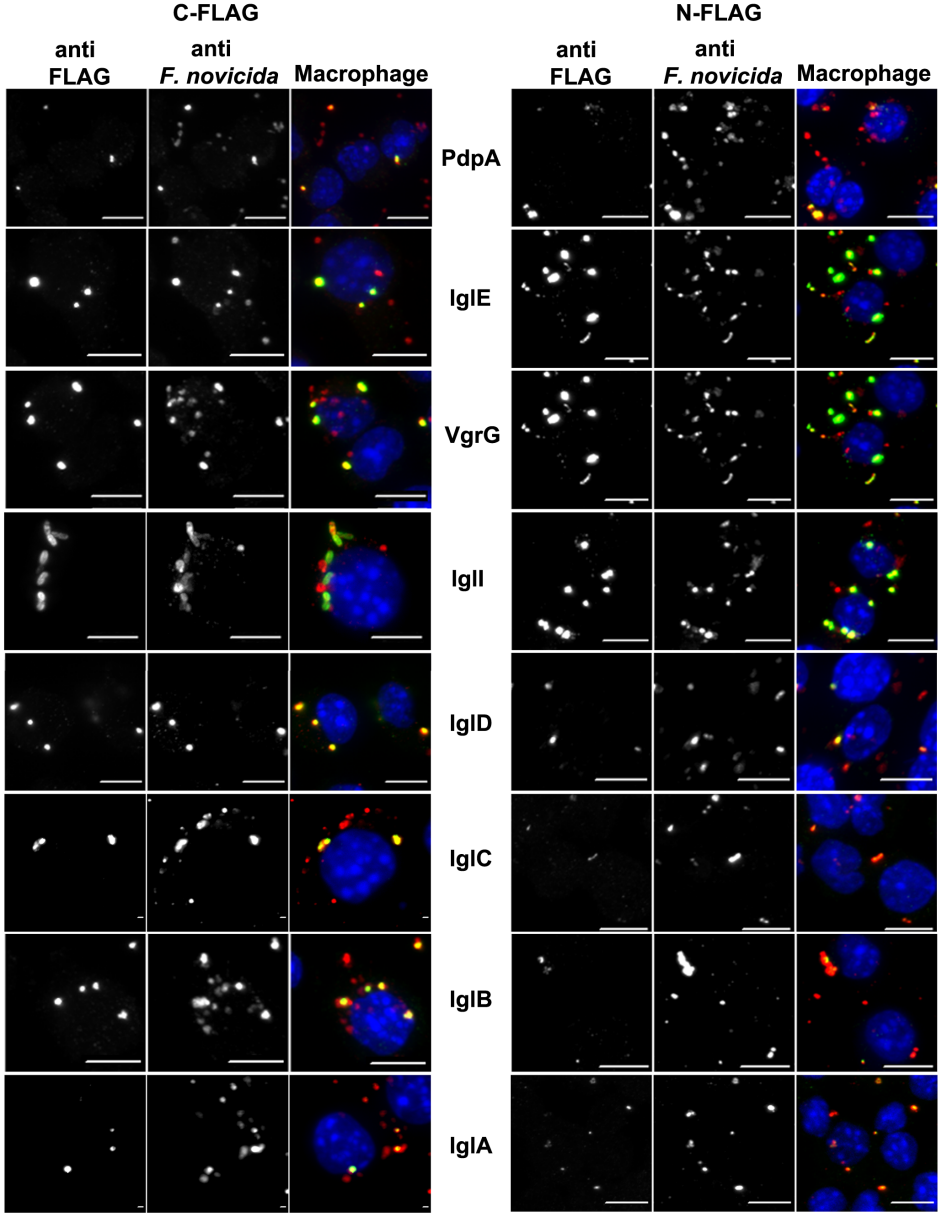
Localization of FPI Proteins within Infected Macrophages. J774 cells were infected with wild type bacteria without a plasmid and wild type containing the *Francisella* expression plasmids that express PdpA, IglABCDEDI and VgrG with either C-term FLAG tag (left) or N-term FLAG tag (right) at 4 h post-infection. Middle columns and the red in the merged right columns indicate bacteria. Left columns and the green in the merged right columns indicate FLAG-tagged proteins. Host cell nuclei are indicated by blue in merged right columns.

**Figure 4:**
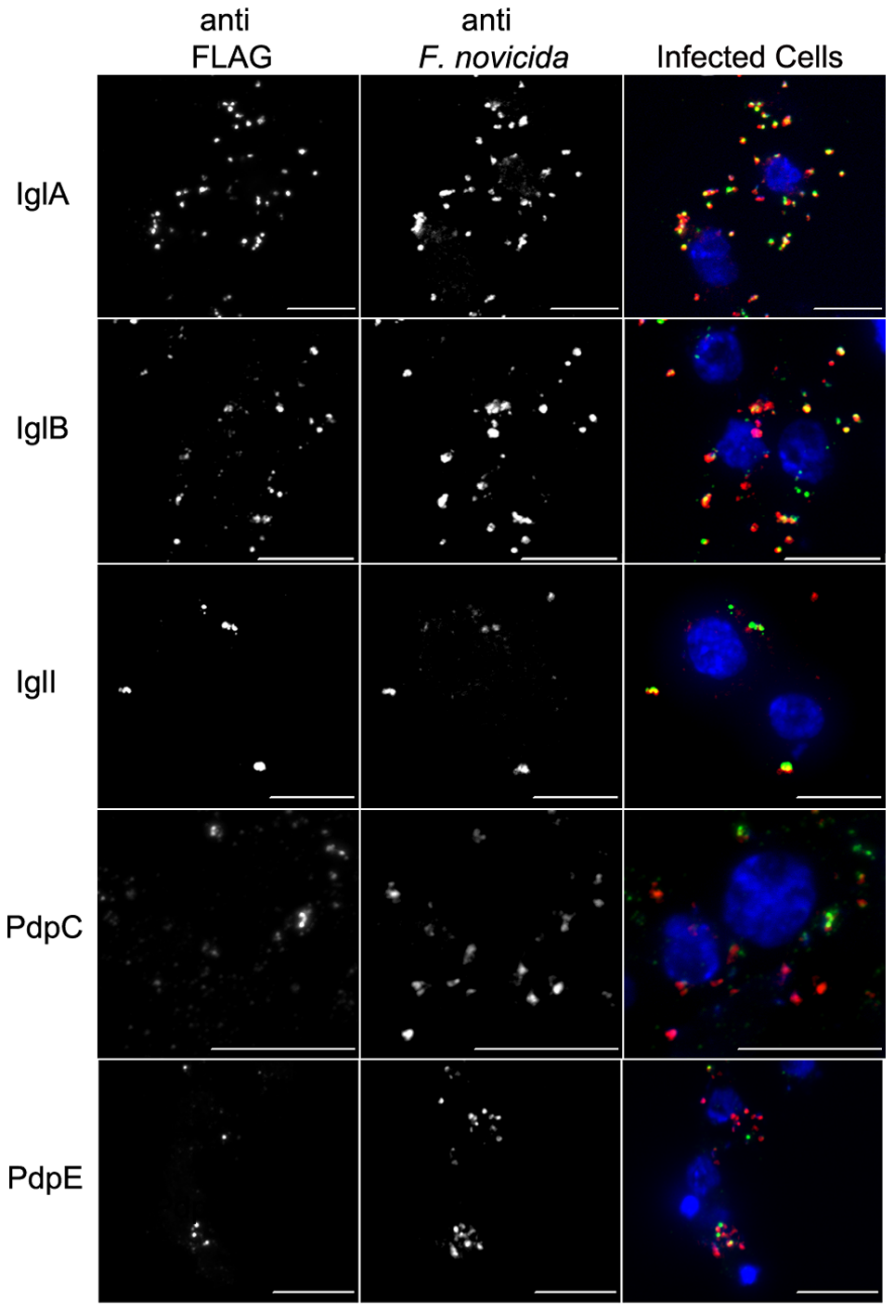
Localization of FPI Proteins During Infection of Sua-1B Cells. J774 cells infected with wild type containing the *Francisella* expression plasmids that express IglA, IglC, IglI, and PdpE for 4 h (A). Sua-1B cells infected with wild type containing the *Francisella* expression plasmids that express IglA, IglC, IglI, and PdpE for 4 h (B). Middle columns and red in the right column indicate bacteria. C-terminally FLAG-tagged proteins are shown in the left columns and are also green in the right column. Host cell nuclei are displayed as blue in the merged, right columns.

FPI proteins with consistent FLAG detection had varying patterns of distribution of FLAG signal when compared to each other within infected murine macrophage-like cells (Fig. 3) and mosquito hemocyte-like cells (Fig. 4). IglA was localized with bacteria (Fig. 3 and Fig. 4). IglCE and VgrG were co-localized with bacteria and also extending beyond, completely surrounding bacteria (Fig. 3 and Fig. 4). IglD and PdpA were also localized with bacteria, and on occasion surrounding the bacteria (Fig. 3 and Fig. 4). PdpC was also detected both co-localized with bacteria and dispersing away from the bacteria (not shown). IglI was distinctly localized to the bacterium, it surrounded the bacterium uniformly (Fig. 3 and Fig. 4). The C-FLAG PdpE was studded around the bacterium, while the N-terminal tagged protein was not detected by immuno-fluorescent microscopy (Fig. S3).

### C-terminal FLAG-tagged FPI Proteins Co-Localization with Bacteria During Cell Infection

**Figure 5:**
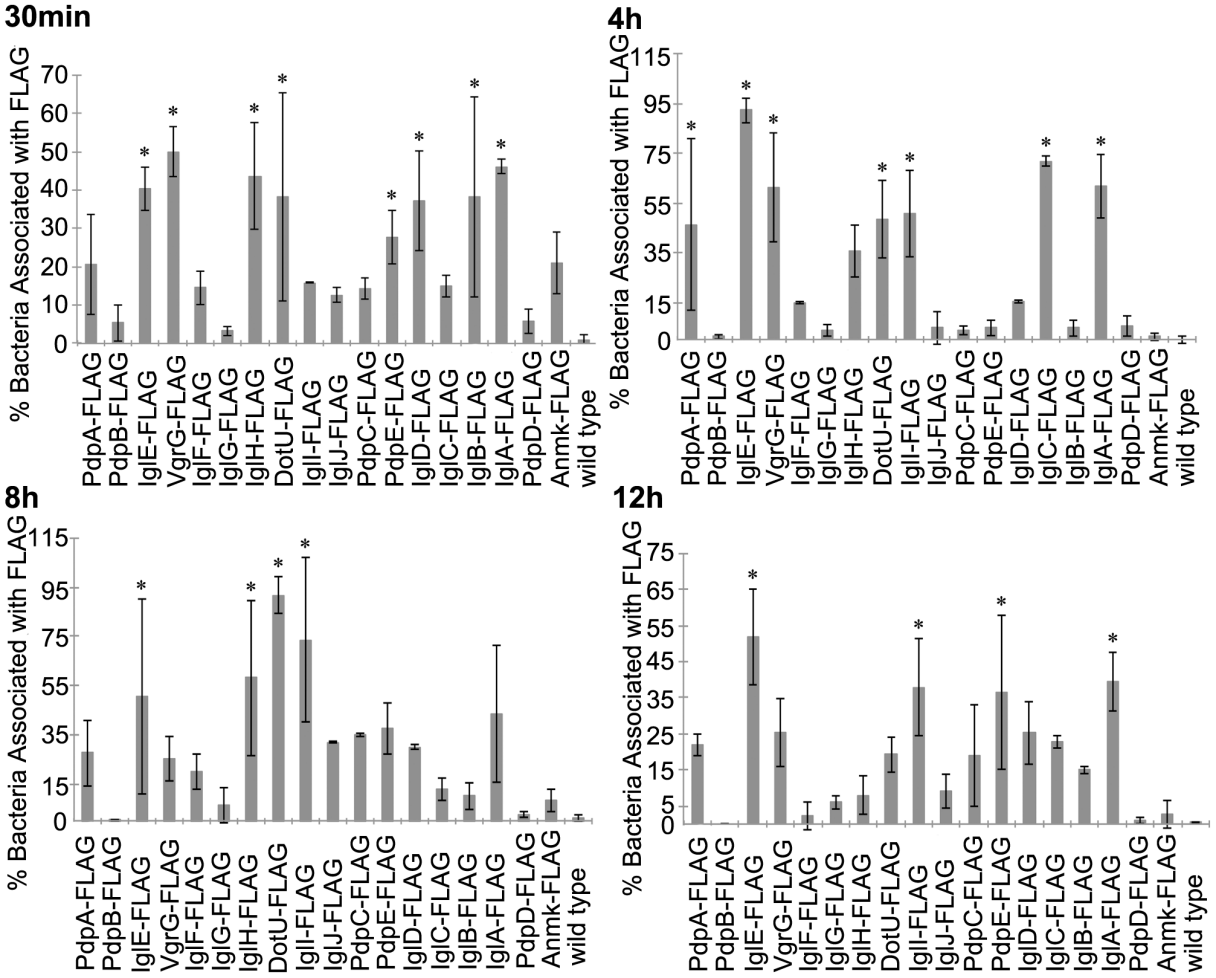
Bacteria Associated with FPI C-tagged Proteins. The percent of bacteria associated with C-term FLAG-tagged FPI proteins within J774 cells at various time-points during infection were determined. This graph represents the mean of three independent experiments. Error bars indicate the standard deviations. Asterisks indicate significance (p≤0.05) and plus signs indicate approaching significance (p = 0.051–0.099) when compared to the no plasmid wild type control.

The localization of FPI-encoded proteins was examined via immuno-fluorescent microscopy of infected murine macrophage-like J774 cells with bacteria expressing C-terminal fusion proteins. The percent of bacteria co-localized with FLAG signal within infected macrophages was determined for all 18 FPI-encoded proteins. At 30 min post-infection, bacteria expressing FLAG-tagged IglABDEH, PdpE, VgrG, and DotU were significantly more often co-localized with fluorescent signal compared to control bacteria not expressing epitope-tagged protein (p≤0.038) (Fig. 5A and Table S2). Bacteria expressing the remaining FLAG-tagged FPI-encoded proteins were not statistically different from the control bacteria at 30 min (p≥0.368) (Fig. 5A and Table S2). Bacteria expressing FLAG-tagged IglACEI, PdpA, VgrG, and DotU all had significantly more bacteria co-localized with fluorescent signal compared to control bacteria not expressing epitope-tagged protein (p≤0.008) at 4 h post-infection (Fig. 5B and Table S2). Also at 4 h, bacteria expressing tagged IglH were approaching statistical significance when compared to the control (p = 0.054) (Fig. 5B and Table S2). The bacteria expressing the rest of the FPI-tagged proteins were not different from control bacteria, when examining the bacteria co-localized with FLAG signal at 4 h (p≥0.814) (Fig. 5B and Table S2). 8 h into infection, bacteria expressing IglEHI and DotU had significantly more bacteria co-localized with fluorescent signal than the control bacteria (p≤0.029) (Fig. 5C and Table S2). Also at 8 h, bacteria expressing IglA had 55% of bacteria co-localized with FLAG signal, approaching statistical significance when compared to the control (p = 0.054) (Fig. 5C and Table S2). The other FLAG-tagged FPI-encoded proteins were not different from the controls (p≥0.274) (Fig. 5C and Table S2). More bacteria were co-localized with fluorescent signal from tagged IglE, DotU, PdpE, and IglA (p≤0.012) with bacteria expressing those tagged proteins compared to control bacteria at 12 h, while bacteria expressing the other FPI-tagged proteins were not different than the control bacteria (p≥0.129) (Fig. 5D and Table S2).

### C-term FLAG-tagged FPI Protein Localization within Infected Cells

**Figure 6:**
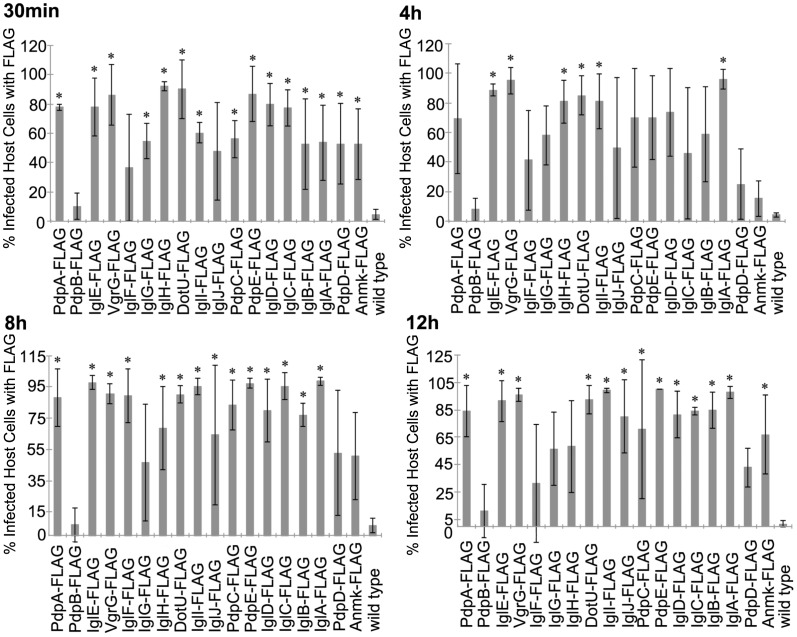
Infected Macrophages with FPI C-tagged Proteins. The percent of infected macrophages containing C-term FLAG-tagged FPI proteins within J774 cells at various time-points during infection were determined. This graph represents the mean of three independent experiments. Error bars indicate the standard deviations. Asterisks indicate significance (p≤0.05) and plus signs indicate approaching significance (p = 0.051–0.099) when compared to the no plasmid wild type control.

The localization of C-term FLAG-tagged FPI proteins were alternatively assessed by calculating the percent of infected cells containing fluorescent signal to account for proteins that were secreted into the infected cells but did not co-localize with the bacteria expressing the epitope tagged proteins. Within 30 min of infection, the cells infected with bacteria expressing tagged IglABCDEHI, PdpACDE, VgrG, DotU, and Amnk all had significantly more infected cells containing fluorescent signal when compared to the control cells infected with bacteria not expressing FLAG-tagged proteins (p≤0.036) (Fig. 6A and Table S2). Also at 30 min into infection, the cells infected with bacteria expressing tagged PdpB and IglF were not different from cells infected with control bacteria (p≥0.232) (Fig. 6A and Table S2). At 4 h post infection, cells infected with bacteria expressing tagged IglEHI, VgrG, and DotU, all had significantly more infected cells containing fluorescent signal (p≤0.029) than the cells infected with bacteria not expressing FLAG-tagged proteins. However, the cells infected with tagged IglBCDFGJ, PdpABCE, and Anmk were not significantly different from cells infected with bacteria not expressing FLAG-tagged proteins (Fig. 6B and Table S2). When comparing the amount of infected cells containing FLAG signal at 8 h after infection, the infected cells that expressed IglABCDEFGI, PdpACE VgrG, and DotU had significantly more infected cells containing FLAG signal (p≤0.009). While the cells infected with bacteria expressing tagged IglG, PdpBD and Anmk were not different from the control cells containing bacteria not expressing FLAG-tagged proteins (p≥0.142) (Fig. 6C and Table S2). More infected cells contained FLAG signal at 12 h within cells infected with bacteria expressing tagged PdpACE, IglABCDEJ, VgrG, DotU, and Anmk (p≤0.042). Additionally at 12 h, cells infected with bacteria expressing FLAG-tagged PdpBD and IglFGH were not statistically different (p≥0.103) when compared to cells infected with bacteria not expressing FLAG-tagged proteins (Fig. 6D and Table S2).

### Bacteria Co-Localization with N-terminal FLAG-tagged FPI Proteins During Cell Infection

**Figure 7:**
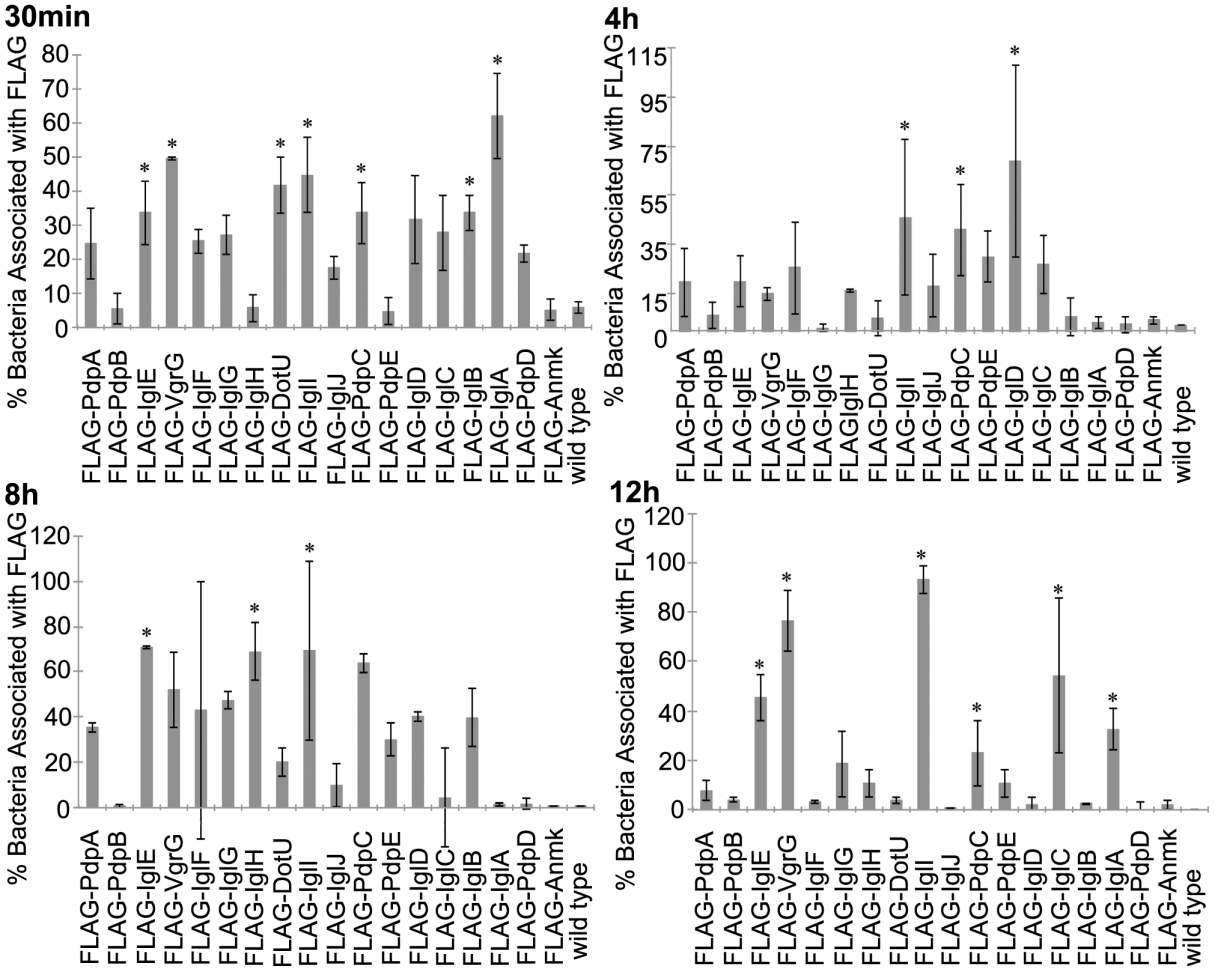
Bacteria Associated with FPI N-tagged Proteins. The percent of bacteria associated with N-term FLAG-tagged FPI proteins within J774 cells at various time-points during infection were determined. This graph represents the mean of three independent experiments. Error bars indicate the standard deviations. Asterisks indicate significance (p≤0.05) and plus signs indicate approaching significance (p = 0.051–0.099) when compared to the no plasmid wild type control.

The localization of FPI-encoded proteins was also examined via immuno-fluorescent microscopy of infected murine macrophage-like cells with bacteria expressing N-terminal fusion proteins in order to assess effects of tags on the termini of FPI encoded proteins. The percent of bacteria co-localized with FLAG signal within infected macrophages was determined for all 18 FPI proteins with a N-terminus FLAG tag. At 30 min post-infection, bacteria expressing FLAG-tagged IglEI, VgrG, DotU, and PdpC had significantly more bacteria co-localized with FLAG signal compared to bacteria not expressing epitope-tagged protein (p≤0.047 (Fig. 7A and Table S2). Also at 30 min, bacteria expressing tagged IglD were approaching statistical significance with the amount of bacteria co-localized with FLAG-tagged protein when compared to the control bacteria (p = 0.072) (Fig. 7A and Table S2). The bacteria expressing the other tagged proteins at 30 min were not statistically different from the control bacteria not expressing tagged protein (p≥0.166) (Fig. 7A and Table S2). Bacteria expressing tagged IglID and PdpC had significantly more bacteria co-localized with FLAG signal compared to cells infected with control bacteria not expressing epitope-tagged protein (p≤0.021) at 4 h post-infection (Fig. 7B and Table S2). Additionally at 4 h into infection, bacteria expressing the rest of the FPI-tagged proteins were not different from control bacteria when examining the bacteria co-localized with FLAG signal (p≥0.347) (Fig. 7B and Table S2). Bacteria expressing tagged IglEI and DotU had significantly more bacteria co-localized with FLAG signal than cells infected with the control bacteria at 8 h post infection (p≤0.037) (Fig. 7C and Table S2). Bacteria expressing the other FPI proteins were not different from the control (p≥0.161) (Fig. 7C). More bacteria were co-localized with FLAG signal from tagged VgrG, DotU, IglACEI, and PdpC (p≤0.032) when compared to control bacteria at 12 h, while bacteria expressing the other FPI-tagged proteins were not different than the control bacteria (p≥0.115) (Fig. 7D and Table S2).

### N-term FLAG-tagged FPI Protein Localization within Infected Cells

**Figure 8:**
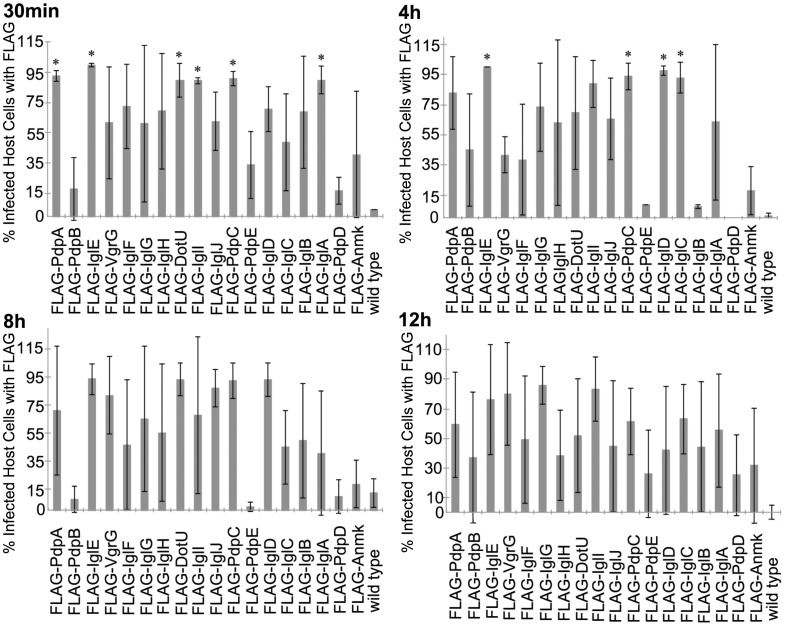
Infected Macrophages with FPI N-tagged Proteins. The percent of infected macrophages containing N-term FLAG-tagged FPI proteins within J774 cells at various time-points during infection were determined. This graph represents the mean of three independent experiments. Error bars indicate the standard deviations. Asterisks indicate significance (p≤0.05) and plus signs indicate approaching significance (p = 0.051–0.099) when compared to the no plasmid wild type control.

Similar to the analysis of C-terminally tagged proteins, the localization of FPI proteins with N-term FLAG tags were also assessed by calculating the percent of infected cells containing FLAG signal. At 30 min into the infection, cells infected with bacteria expressing tagged PdpAC, IglAEI, DotU, and PdpC all had significantly more infected cells containing FLAG signal when compared to control cells infected with bacteria not expressing FLAG-tagged proteins (p≤0.008) (Fig. 8A and Table S2). Also at 30 min, cells infected with bacteria expressing tagged IglBCDFGHJ PdpB, VgrG, and PdpDE, and Amnk were not significant (p≥0.135) (Fig. 8A and Table S2). Cells infected with bacteria that expressed tagged IglCDE and PdpC all had significantly more infected cells containing FLAG signal (p≤0.045) than cells infected with bacteria not expressing FLAG-tagged proteins at 4 h post-infection (Fig. 8B and Table S2). Additionally at 4 h post infection, the cells infected with bacteria expressing tagged PdpABD, IglABIFGHJ, VgrG, DotU, and Anmk were not significantly different from the cells infected with bacteria not expressing FLAG-tagged proteins (p≥0.173) (Fig. 8B and Table S2). When comparing the amount of infected cells containing FLAG signal at 8 h after infection, the cells infected with bacteria that expressed tagged FPI proteins were not different (p≥0.132) than infected cells containing bacteria not expressing FLAG-tagged proteins (Fig. 8C and Table S2). None of the infected cells were significant for containing FLAG signal in cells infected (p≥0.105) when compared to cells infected with bacteria not expressing FLAG-tagged proteins at 12 h into the infection (Fig. 8D and Table S2).

### IglC Localization was Dependent on PdpB

**Figure 9:**
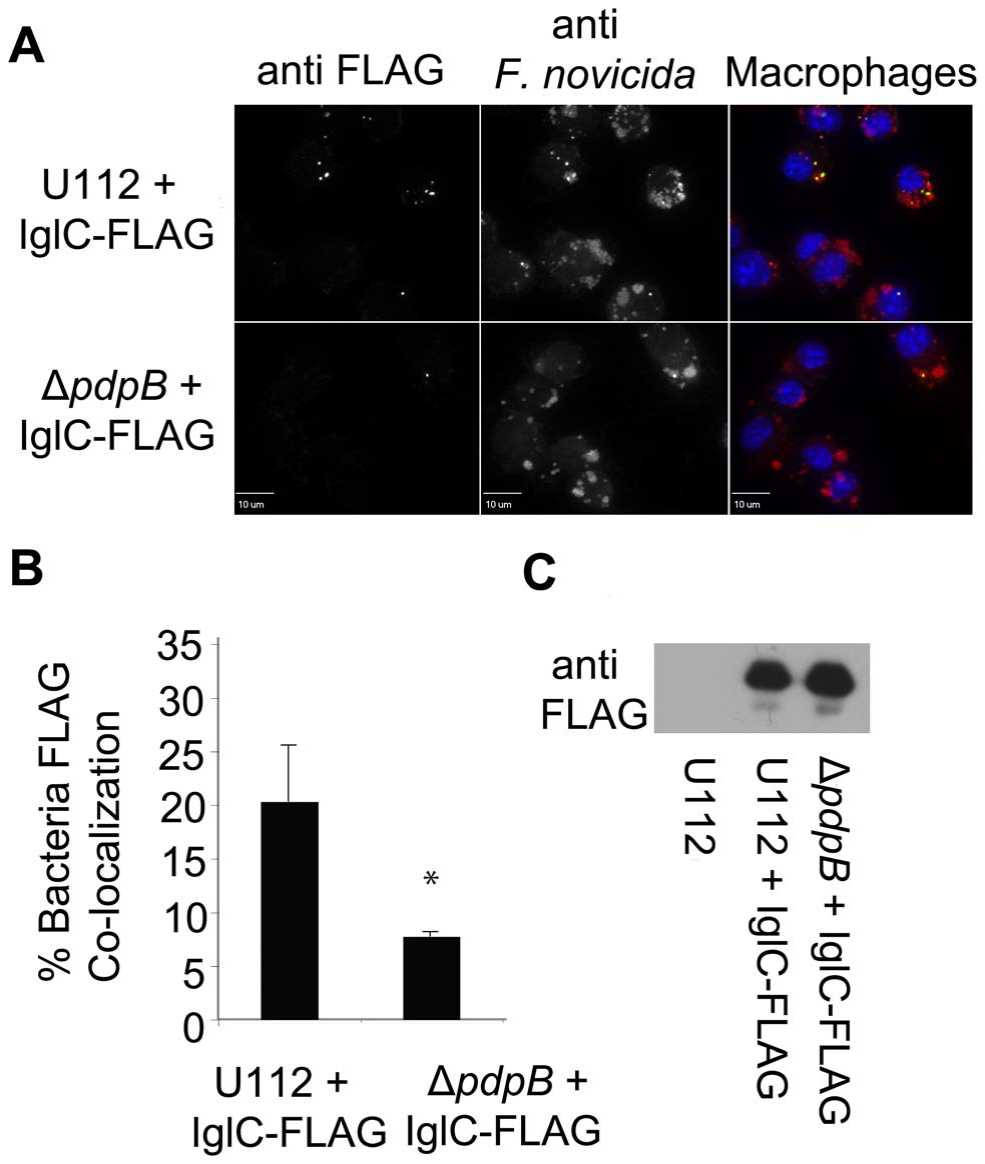
IglC Secretion was Dependent on T6SS. Microscopy of IglC-FLAG expressed in both wild type and Δ*pdpB* strains of U112 within J774 cells at 4 h post infection (A). Automated image analysis of bacterial cells associated with FLAG signal in wild type and Δ*pdpB* backgrounds shown in (B). Each bar represents the mean from three independent experiments of the percent of bacteria associated with FLAG signal, and error bars represent the standard deviation. An asterisk indicates the value was significantly different from that of the wild type in a Two-tailed t test (p≤0.05). Western blot shows FLAG expression of IglC-FLAG in wild type and Δ*pdpB* strains of U112 (C).

Less FLAG was detected when IglC was expressed by the Δ*pdpB* mutant when compared to expression of tagged IglC by wild type bacteria (Fig. 9). Over 20% of wild type bacteria were associated with IglC, while less than 7% of Δ*pdpB* bacteria were associated with IglC (Fig. 9B). The differences between IglC association with wild type or Δ*pdpB* mutant bacteria were statistically significant. IglC was examined by Western blot to determine if the mutant expressed the tagged proteins. IglC-FLAG was expressed at similar levels in the wild type and Δ*pdpB* mutant backgrounds (Fig. 9C). Therefore, extracellular co-localization of IglC with bacteria was dependent on the T6SS.

## Discussion

Genes within the FPI are required for a T6SS, intracellular growth, and virulence [13], [24], [25], [32], [33]. Many of the FPI-encoded proteins are part of a T6SS, therefore we hypothesized that some of the FPI encoded proteins would be directed for secretion by that secretion system, such as effector proteins or chaperones [11], [33]. A recent study that utilized a fusion Temoniera (TEM) β-lactamase reporter in LVS identified IglCEFIJ, PdpAE, and VgrG as secreted proteins, and determined that secretion is dependent on the core components of the T6SS: IglCG, DotU, and VgrG [11]. Also within that same study, only IglCE and PdpAE are secreted from *F. novicida*, suggesting differences in the secretion of FPI proteins among subspecies of *Francisella* [11]. In another study a CyaA reporter was used to show IglI and VgrG are secreted in both LVS and *F. novicida* [33].

Although these studies were the first to identify secreted FPI-encoded proteins, there were some limitations to the tools used in those experiments. The TEM β-lactamase reporters have adverse effects including low levels of protein expression; therefore results were only based on one time point at 18 h post-infection [11]. In addition to low expression, the TEM β-lactamase expresses functionless proteins that are unable to complement an intracellular growth phenotype [11]. The TEM β-lactamase assay was used in a *F. novicida* β-lactamase mutant because *F. novicida* possesses native β-lactamases that interfered with the fusion tag [11].

In the present study, we examined the localization of FPI proteins in *F. novicida* during infection of macrophage-like cells. We used *Francisella* expression plasmids that express all 18 of the FPI-encoded proteins from *F. novicida* with a C-terminal epitope FLAG tag as well as a N-terminal epitope FLAG tag. The *Francisella* expression plasmids were the first tools described for *Francisella novicida* that have epitope tags for both termini for the entire set of FPI proteins. Some secreted proteins possess a secretion signal on either the N- or C-terminus [16]. Consequently, adding amino acids at either end may block the secretion signal, disrupting the protein's localization and function. It was unlikely that both the N- and C- termini of the proteins were required for localization. Therefore, the *Francisella* expression plasmids were developed to contain epitope FLAG tags at both the N- and C- termini. Moreover, the triple FLAG tag is short; it was less likely to alter protein folding and function. Triple repeating sequences increase affinity of FLAG monoclonal antibodies and reduce background in Western blotting, immuno-fluorescence microscopy, and many other commercially available biochemical products specific for localizing the FLAG tag sequence. However, there were also some disadvantages associated with the *Francisella* expression plasmids. Adding the triple FLAG tag does increase the molecular weight and could alter protein processing as evident by altered migration pattern in SDS gels (Fig. 1 and Fig. S2). These proteins encoded by the plasmids were constitutively expressed, which may lead to over expression and could also explain the apparent altered processing for some proteins. Additionally the FPI blots were performed with similar amounts of bacteria and not adjusted for optimal visualization of individual proteins, which lead to over exposure of some proteins (Fig. 1). The lack of a wild type PdpC band visible with the PdpC antibody could be explained by low expression levels of native PdpC compared to the overexpression of PdpC-FLAG. Also it should be noted that different antibodies were used for detection of PdpC; the FLAG-tagged proteins contain three repeats of the FLAG sequences, which increases the avidity of the FLAG antibodies compared to the antibody raised against native PdpC (Fig. S2B).

To help discern the functions of FPI-encoded proteins, their localization within host cells were examined via microscopy. There are limitations associated with microscopy. Microscopy permeabilization techniques can result in false positives due to cell death or leakiness of membranes. In this study we used saponin to permeate host cell membranes, while leaving the bacterial cell membranes intact [30].

If the localization of a FPI protein was dependent on the expression of other proteins at a specific time, then we would have detected it because we examined four different time points during infection. These time points were chosen according to *Francisella*'*s* intracellular life cycle [34], [35]. By 30 min post-infection, internalized bacteria escape the phagosome and enter the host cell's cytoplasm [20], [31]. Bacteria replicate intracellularly by four and 8 h bacteria post-infection [35]. By 12h post-infection bacteria manipulate host cells by avoiding immune responses and initiating autophagy [37]. However, since the plasmids constitutively express proteins inferences cannot be made on the timeline of secretion under natural transcriptional control.

In this study the secretion and localization of FPI tagged proteins during an infection of a macrophage-like cell line was determined by assessing the amount of fluorescence per cell. First, we generated a complete set of plasmids that contain either N- or C-terminal tags. These plasmids were used to examine the entire FPI for potentially secreted proteins, and internal controls within the FPI were used for this study. IglE is described as an outer-membrane protein, PdpB is an inner-membrane protein, and several FPI proteins have previously been identified as secreted in *F. tularensis* [11], [13], [33]. As an initial screen of the entire FPI, we hope this study inspires future investigations that further characterize individual FPI proteins and their secretion pattern and localization within infected cells.

Using the *Francisella* expression plasmids in *F. novicida* IglABCDEFGHIJ, PdpACE, DotU, and VgrG were localized within macrophage-like cells on the outside of bacterial cell membranes. Proteins that were consistently detected in the cytoplasm of host cells were IglABCDEI and VgrG. There were high degrees of variation observed among time points, in the localization of proteins within infected host cells, among the N-terminal tagged proteins at, and between different tags of the same proteins. The detection of IglFGHJ, PdpACE, and DotU varied depending on time point. Since these proteins were constitutively expressed inferences based on time would not be valid. Although if some of the FPI proteins require the expression of other proteins for their delivery, secretion, or localization this may have been missed with only examining one time point. The variance in the cellular analyses can be explained by the fact that more area was assessed in the cell level analysis increasing total non-specific background fluorescence compared to bacterial association. Additionally, it was not possible to address how bacteria that were co-localized with FLAG were dispersed among infected cells. In addition to the increase in background signal in the analysis of host cells infected with FLAG, the variation among the N-tagged proteins can be explained by the fact that proteins are translated from N-terminus to C-terminus; therefore, the additional amino acid sequences from the FLAG tag may have an affect on folding and protein stability, however these proteins were able to restore the growth phenotype (Fig. 2). It was also not surprising that N-terminally tagged PdpE was not expressed, as PdpE is predicted to have an export signal sequence on the N-terminus [11]. In addition IglE also may possess an N-terminal signal and is an outer-membrane protein [11].

Since the C-terminal and the N-terminal-flagged proteins were individually analyzed for each protein, the expression levels of the two variants were statistically compared for correlation (Fig. S4). The two tags were significantly correlated at half of the time points; overall there was always a positive correlation trend. Reasons that detection of N- and C-terminally tagged proteins were not always correlated could include effects from the tag such as alterations in expression and processing, post translation modifications, masking transportation of proteins, or stability. To avoid artifacts from tagging one terminus of the individual FPI proteins, this study used two tags and examined several time points. It should also be noted that for a given protein there was also a wide variation from one time point to another (Fig. 5–8). This is not surprising since the stabilities of these proteins are not known and previous studies of stability of pathogen proteins in host cells can be altered by tagging effects or degraded by host cell [37].

Some proteins, while expressed, were not detected in the cytoplasm of host cells. The lack of detection of fluorescence signal for PdpBD and Anmk possessing FLAG tags were similar to that of wild type cells in bacteria, and within infected host cells. This suggests PdpBD and Anmk were not secreted from bacteria, which is consistent with other studies [13], [38]. These non-secreted proteins were expressed at similar levels compared to most of the secreted proteins (Fig. 1). Our inability to detect PdpB confirms the appropriateness of using saponin-permeabilized cells to detect FLAG epitopes outside of bacteria while leaving the bacterial cell wall intact [30]. Previous fractionation of *F. novicida* expressing FLAG-tagged PdpB show this PdpB localized to the inner-membrane [13]. The current model for the T6SS in *Francisella* suggests PdpB is a transmembrane anchor protein, which spans the inner-membrane with parts extending into the periplasmic space [13]. DotU is an inner-membrane component of the secretion system of *Francisella* and all T6SS's where it interacts with PdpB [13], [38]. Solubility properties have identified DotU as predominantly membrane-associated, partially soluble, and localized to the inner-membrane and periplasmic space where it stabilizes the secretion system [13], [39]. The localization of DotU has not been visualized before; it is interesting that in this study, microscopy detected DotU as extracellular. DotU could be temporarily exposed to the extracellular space of bacterial cells during the contraction of the tube of the secretion system as proteins were secreted. Also the extracellular localization of DotU could be from effects of the FLAG tag.

In the current model of the T6SS in *Francisella novicida* as described by de Bruin, the inner-tube of the T6SS is speculated to be a polymer of IglC, which lies within the IglA and IglB polymer that contracts and drives IglC through the host cell membrane [13]. This contraction of IglAB could temporarily expose components of the secretion system (IglABC, DotU, and potentially other proteins) to extracellular staining. Also, IglA-IglB polymers span both the inner- and outer-membrane of *Francisella*, and thus were exposed extracellularly but not necessarily secreted. VgrG and PdpE were located on the point of the secretion channel-forming tube and would therefore appear outside of bacteria, as shown in this study [13].

Several secreted proteins were identified in this study that have previously been identified as secreted from LVS, including PdpAE, IglCEFIJ, and VgrG [11], [33], [39]. This study also identified DotU, IglABDGH, and PdpC as being localized to the outside of bacteria within infected host cells. Fractionation of *F. novicida* show IglABCD localized in all fractions of the bacterial cell [13], which supports their detection, outside of bacteria within macrophages. IglH, and PdpC might be secreted proteins, as it is not clear whether these proteins were secreted, localized to the outer-membrane of *Francisella*, or temporarily localized to the outer-membrane as components of the secretion system during transport of other secreted proteins. An alternative explanation is their detection in this study is leakage due to over expression from the *Francisella* expression plasmids. Detection observed in microscopy is not likely from dying cells that leak tagged proteins; since we have shown these tagged, plasmid-expressed proteins restored the intracellular growth phenotype (Fig. 2). In addition, PdpB, a protein localized to the inner bacterial membrane was not detected in microscopy while it was expressed (Fig. 1) and restored growth (Fig. 2). If leakage were a systematic problem with this study, we would expect PdpB to be detected despite its localization to the inner-membrane. However, we cannot exclude this possibility for other proteins. In any type of secretion assay, leakage from dead or dying bacteria is always a possibility that has to be considered in data interpretation.

To further examine localization of IglC, the IglC-FLAG plasmid was transformed into a Δ*pdpB* strain to test if localization was dependent on the T6SS. PdpB is homologous to IcmF, which is an inner-membrane component of the T6SS in *V. cholera* and is required for the secretion of Hcp [14]. PdpB was not detected through microscopy because it is an inner-membrane protein of *Francisella* [13]. The co-localization of IglC-FLAG with bacterial cells was significantly lower in Δ*pdpB* bacteria compared to wild type cells (Fig. 9B). The expression of IglC was examined to determine if the mutant was expressing IglC-FLAG, and both wild type and Δ*pdpB* expressed IglC at similar levels (Fig. 9C).

The relevance of the findings may not be generally applicable to other, virulent subspecies of *Francisella* since there are previous data showing that the secretion patterns differ between *F. novicida* and LVS [11], [33]. Although, due to the similarities of the secreted proteins between LVS and *F. novicida*, this study confirms *F. novicida* as a valuable model to study the molecular mechanism employed by *F. tularensis* during infection of host cells.

This study describes the development of genetic tools to assess and elucidate the function of the complete set of FPI encoded proteins. These genetic tools include plasmids that contain an entire set of both N- and C-terminus epitope triple FLAG-tagged FPI genes that express FPI proteins. Western blotting of bacterial lysates reveals expression of 35 full-length epitope tagged FPI proteins. The *Francisella* expression plasmid expresses full-length functional proteins that restore the intramacrophage growth phenotype in respective mutants. Therefore the *Francisella* expression plasmids were genetically viable tools that can be used to further understand the intracellular life cycle of *F. tularensis* and elucidate potential intervention strategies. Overall these plasmids will contribute to a better understanding of the molecular mechanisms involved in the intracellular life cycle of *F. tularensis.*

## Supporting Information

Figure S1 ***Francisella*** **Expression Plasmids.** Representative diagram of the *Francisella* expression plasmids, pKH4 containing *iglC*. with a C-terminal FLAG tag and pKH46 containing *iglC* with a N-terminal FLAG tag, are shown as examples of all 36 plasmids. All of the *Francisella* expression plasmids contain a groE promoter, a multiple cloning site (MCS), triple FLAG epitope tag, antibiotic cassettes, and an origin of replication. The MCS shows restriction enzyme sites used for insertion of FPI genes. Each of the FPI genes was individually inserted where *iglC* is depicted in the diagram. Arrows represent the direction of transcription and size of gene products.
(TIF)

Figure S2 **Native and FLAG-tagged FPI Proteins.** Western blot of *F. novicida* U112 wild type and U112 expressing the respective C-terminally tagged FPI proteins from the *Francisella* expression plasmid are labeled above each lane. (A) 15% gel with IglAC, (B) 8% gel with PdpAC. IglAC and PdpAC proteins were detected with polyclonal rabbit anti IglAC and PdpAC. FLAG-tagged proteins are detected with monoclonal mouse anti FLAG, goat anti mouse conjugated HRP, and chemiluminescent substrate.
(TIF)

Figure S3 **Three-dimensional Reconstruction of PdpE.** Three-dimensional reconstructions were comprised from a series of images that were taken through the macrophage cell infected with wild type containing *Francisella* expression plasmids. Bacteria in red, FLAG-tagged protein in green, and host cell nuclei in blue.
(MOV)

Figure S4 **N- and C-tag Correlation.** The data for N- and C-tagged proteins in both bacterial and cellular analyses were plotted against each other for each protein, at each time point. The data were subjected to a Spearman correlation tests. A best-fit trend line was inserted along with the slope, R^2^ values, and the P value. Asterisks indicate significance (p≤0.05) of the Spearman's test for correlation. Specific analysis and times points are indicated on graphs.
(TIF)

Table S1 **Molecular weights of FPI proteins.** The molecular weights of *F. novicida* FPI encoded proteins.
(DOCX)

Table S2 **Means of FLAG with bacteria and FLAG within cells and significance.** Each of the FPI proteins were examined for their localization with bacteria or within infected host cells, which is indicated as Bacterial or Cellular in the analysis column. Within each analysis proteins were examined via the FLAG tag on the N-terminus and the C-terminus. Values indicate the mean percentage of bacterial-FLAG co-localization or the mean percentage of infected cells containing FLAG from 3 independent experiments. Significance was determined with a left sided Dunett's test, * p<0.05, **p<0.001, and ***p<0.0001.
(DOCX)

## References

[1] Gal-Mor O, Finlay BB (2006) Pathogenicity islands: a molecular toolbox for bacterial virulence. Cell Microbiol 8(11): 1707–1719. 16939533 10.1111/j.1462-5822.2006.00794.x

[2] Galan JE, Wolf-Watz H (2006) Protein delivery into eukaryotic cells by type III secretion machines. Nature 444(7119): 567–573. 17136086 10.1038/nature05272

[3] Mattoo S, Lee YM, Dixon JE (2007) Interactions of bacterial effector proteins with host proteins. Curr Opin Immunol 19(4): 392–401. 17662586 10.1016/j.coi.2007.06.005

[4] Nano FE, Zhang N, Cowley SC, Klose KE, Cheung KK, et al (2004) A *Francisella tularensis* pathogenicity island required for intramacrophage growth. J Bacteriol 186(19): 6430–6436. 15375123 10.1128/JB.186.19.6430-6436.2004 PMC516616

[5] Owen CR, Burker EO, Jellison WL, Lackman DB, Bell JF (1964) Comparative studies of *Francisella tularensis* and *Francisella novicida* . J Bacteriol 87: 676–83. 14127585 10.1128/jb.87.3.676-683.1964 PMC277070

[6] Gray CG, Cowley SC, Cheung KK, Nano FE (2002) The identification of five genetic loci of *Francisella novicida* associated with intracellular growth. FEMS Microbiol Lett 215(1): 53–56. 12393200 10.1111/j.1574-6968.2002.tb11369.x

[7] Tempel R, Lai XH, Crosa L, Kozlowicz B, Heffron F (2006) Attenuated *Francisella novicida* transposon mutants protect mice against wild type challenge. Infect Immun 74(9): 5095–5105. 16926401 10.1128/IAI.00598-06 PMC1594869

[8] Maier TM, Havig A, Casey M, Nano FE, Frank DW, et al (2004) Construction and characterization of a highly efficient *Francisella* shuttle plasmid. Appl Environ Microbiol 70(12): 7511–7519. 15574954 10.1128/AEM.70.12.7511-7519.2004 PMC535190

[9] Nix EB, Cheung KK, Wang D, Zhang N, Burke RD, et al (2006) Virulence of *Francisella* spp. in chicken embryos. Infect Immun 74(8): 4809–4816. 16861669 10.1128/IAI.00034-06 PMC1539577

[10] Santic M, Molmeret M, Barker JR, Klose KE, Dekanic A, et al (2007) A *Francisella tularensis* pathogenicity island protein essential for bacterial proliferation within the host cell cytosol. Cell Microbiol 9(10): 2391–2403. 17517064 10.1111/j.1462-5822.2007.00968.x

[11] Bröms JE, Meyer L, Sun K, Lavander M, Sjöstedt A (2012) Unique substrates secreted by the type VI secretion system of *Francisella tularensis* during intramacrophage infection. PLOS ONE 7(11).. 10.1371/journal.pone.0050473 PMC3502320 23185631

[12] de Bruin OM, Ludu JS, Nano FE (2008) The *Francisella* pathogenicity island protein IglA localizes to the bacterial cytoplasm and is needed for intracellular growth. BMC Microbiol 7: 1. 10.1186/1471-2180-7-1 PMC1794414 17233889

[13] de Bruin OM, Duplantis BN, Ludu JS, Hare RF, Nix EB, et al (2011) The biochemical properties of the *Francisella* pathogenicity island (FPI)-encoded proteins IglA, IglB, IglC, PdpB, and DotU suggest roles in type VI secretion. Microbiol 157: 3483–3491. 10.1099/mic.0.052308-0 PMC3352279 21980115

[14] Barker JR, Chong A, Wehrly TD, Yu JJ, Rodriguez SA, et al (2009) The *Francisella tularensis* pathogenicity island encodes a secretion system that is required for phagosome escape and virulence. Mol Microbiol 74(6): 1459–1470. 20054881 10.1111/j.1365-2958.2009.06947.x PMC2814410

[15] Bingle LE, Bailey CM, Pallen MJ (2008) Type VI secretion: a beginner's guide. Curr Opin Microbiol 11(1): 3–8. 18289922 10.1016/j.mib.2008.01.006

[16] Filloux A, Hachani A, Bleves S (2008) The bacterial type VI secretion machine: yet another player for protein transport across membranes. Microbiol 154(2008): 1570–1583. 10.1099/mic.0.2008/016840-0 18524912

[17] Bladergroen MR, Badelt K, Spaink HP (2003) Infection-blocking genes of a symbiotic *Rhizobium leguminosarum* strain that are involved in temperature-dependent protein secretion. Mol Plant Microbe Interact 16(1): 53–64. 12580282 10.1094/MPMI.2003.16.1.53

[18] Pukatzki S, Ma AT, Sturtevant D, Krastins B, Sarracinot D, et al (2006) Identification of a conserved bacterial protein secretion system in *Vibrio cholerae* using the *Dictyostelium* host model system. Proc Natl Acad Sci U S A 103(5): 1528–1533. 16432199 10.1073/pnas.0510322103 PMC1345711

[19] Lindgren H, Golovliov I, Baranov V, Ernst RK, Telepnev M, et al (2004) Factors affecting the escape of *Francisella tularensis* from the phagolysosome. J Med Microbiol 53(10): 953–958. 15358816 10.1099/jmm.0.45685-0

[20] Santic M, Molmeret M, Klose KE, Jones S, Kwaik YA (2005) The *Francisella tularensis* pathogenicity island protein IglC and its regulator MglA are essential for modulating phagosome biogenesis and subsequent bacterial escape into the cytoplasm. Cell Microbiol 7(7): 969–979. 15953029 10.1111/j.1462-5822.2005.00526.x

[21] Mougous JD, Cuff ME, Raunser S, Shen A, Zhou M, et al (2006) A virulence locus of *Pseudomonas aeruginosa* encodes a protein secretion apparatus. Science 312(5779): 1526–30. 16763151 10.1126/science.1128393 PMC2800167

[22] Schell MA, Ulrich RL, Ribot WJ, Brueggemann EE, Hines HB, et al (2007) Type VI secretion is a major virulence determinant in *Burkholderia mallei* . Mol Microbiol 64(6): 1466–1485. 17555434 10.1111/j.1365-2958.2007.05734.x

[23] Zheng J, Leung KY (2007) Dissection of a type VI secretion system in *Edwardsiella tarda* . Mol Microbiol 66(5): 1192–1206. 17986187 10.1111/j.1365-2958.2007.05993.x

[24] Lindgren M, Bröms J, Meyer L, Golovilov I, Sjöstedt A (2013) The *Francisella tularensis LVS Delta-pdpC* mutant exhibits a unique phenotype during intracellular infection. BMC Microbiol 13(20). 10.1186/1471-2180-13-20 PMC3562505 23356941

[25] Lindgren M, Eneslatt K, Bröms J, Sjöstedt A (2013) Importance of PdpC, IglC, IglI, and IglG for modulation of a host cell death pathway induced by *Francisella tularensis* . Infect Immun 81(6): 2076–2084. 23529623 10.1128/IAI.00275-13 PMC3676040

[26] Read A, Sigrid J, Hueffer K, Gallagher L, Happ G (2008) *Francisella* genes required for replication in mosquito cells. J Med Entomol 45(6): 1108–1116. 19058636 10.1603/0022-2585(2008)45[1108:fgrfri]2.0.co;2

[27] Sambrook J, Fritsch EF, Maniatis T (1989) Molecular cloning: a laboratory manual, 2nd ed. Cold Spring Harbor Laboratory, Cold Spring Harbor, N.Y.

[28] Patel JC, Hueffer K, Lam TT, Galan JE (2009) Diversification of a *Salmonella* virulence protein function by ubiquitin-dependent differential localization. Cell 137: 283–294. 19379694 10.1016/j.cell.2009.01.056 PMC2673707

[29] Tyeryar FJ, Lawton WD (1970) Factors affecting transformation of *Pasteurella novicida.* . J Bacteriol 104(3): 1312–1317. 16559109 10.1128/jb.104.3.1312-1317.1970 PMC248293

[30] Johnson MB, Criss AK (2013) Fluorescence microscopy methods for determining the viability of bacteria in association with mammalian cells. J Vis Exp 5(79). 10.3791/50729 PMC3814296 24056524

[31] Chong A, Wehrly TD, Nair V, Fischer ER, Barker JR, et al (2008) The early phagosomal stage of *Francisella tularensis* determines optimal phagosomal escape and *Francisella* pathogenicity island protein expression. Infect Immun 76(12): 5488–5499. 18852245 10.1128/IAI.00682-08 PMC2583578

[32] Schmerk CL, Duplantis BN, Howard PL, Nano FE (2009) A *Francisella novicida pdpA* mutant exhibits limited intracellular replication and remains associated with the lysosomal marker LAMP-1. Microbiol 155(5): 1498–1504. 10.1099/mic.0.025445-0 PMC2889414 19372155

[33] Bröms JE, Sjöstedt A, Lavander M (2010) The role of the *Francisella tularensis* pathogenicity island in type VI secretion, intracellular survival, and modulation of host cell signaling. Front Microbiol 1(136). 10.3389/fmicb.2010.00136 PMC3109350 21687753

[34] Clemens DL, Lee BY, Horwitz MA (2005) *Francisella tularensis* enters macrophages via a novel process involving pseudopod loops. Infect Immun 73(9): 5892–5902. 16113308 10.1128/IAI.73.9.5892-5902.2005 PMC1231130

[35] Santic M, Molmeret M, Klose KE, Kwaik YA (2006) *Francisella tularensis* travels a novel, twisted road within macrophages. Trends Microbiol 14(1): 37–44. 16356719 10.1016/j.tim.2005.11.008

[36] Checroun C, Wehrly TD, Fischer ER, Hayes SF, Celli J (2006) Autophagy-mediated reentry of *Francisella tularensis* into the endocytic compartment after cytoplasmic replication. Proc Natl Acad Sci U S A 103(39): 4578–4583. 10.1073/pnas.0601838103 PMC1600002 16983090

[37] Kubori T, Galan J (2003) Temporal regulation of *Salmonella* virulence effector function by proteasome-dependent protein degradation. Cell 115: 333–342. 14636560 10.1016/s0092-8674(03)00849-3

[38] Ludu JS, de Bruin OM, Duplantis BN, Schmerk CL, Chou AY, et al (2008) The *Francisella* pathogenicity island protein is PdpD is required for full virulence and associates with homologues of the type VI secretion system. J Bacteriol 190(13): 4584–4595. 18469101 10.1128/JB.00198-08 PMC2446798

[39] Leiman PG, Basler M, Ramagopal UA, Bonanno JB, Sauder, et al (2009) Type VI secretion apparatus and phage tail-associated protein complexes share a common evolutionary origin. Proc Natl Acad Sci U S A 106(11): 4154–4159. 19251641 10.1073/pnas.0813360106 PMC2657435

[40] Robertson G, Child R, Ingle C, Celli J, Norgard M (2013) IglE is an outer membrane-associate lipoprotein essential for intracellular survival and murine virulence of Type A *Francisella tularensis* . Infect Immun 81(11): 4026–4040. 23959721 10.1128/IAI.00595-13 PMC3811846

