# Supplementary figures and images for: Francisella novicida Pathogenicity Island Encoded Proteins Were Secreted during Infection of Macrophage-Like Cells
Source: PLoS One. 2014 Aug 26;9(8):e105773. doi: 10.1371/journal.pone.0105773 (PMC4144950; doi:10.1371/journal.pone.0105773)

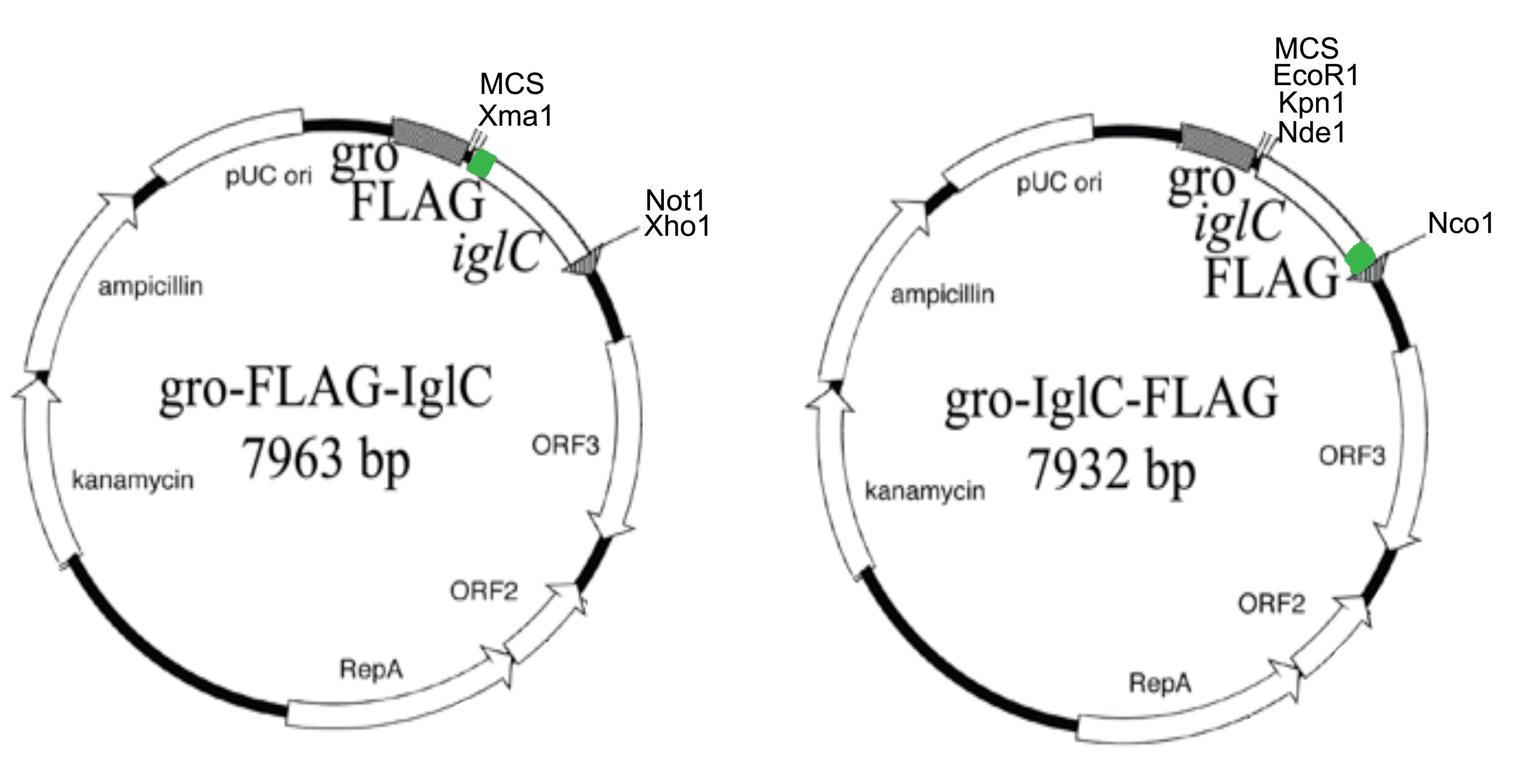

Supplement: Figure S1 — Francisella Expression Plasmids. Representative diagram of the Francisella expression plasmids, pKH4 containing iglC. with a C-terminal FLAG tag and pKH46 containing iglC with a N-terminal FLAG tag, are shown as examples of all 36 plasmids. All of the Francisella expression plasmids contain a groE promoter, a multiple cloning site (MCS), triple FLAG epitope tag, antibiotic cassettes, and an origin of replication. The MCS shows restriction enzyme sites used for insertion of FPI genes. Each of the FPI genes was individually inserted where iglC is depicted in the diagram. Arrows represent the direction of transcription and size of gene products. (TIF) [file pone.0105773.s001.tif]

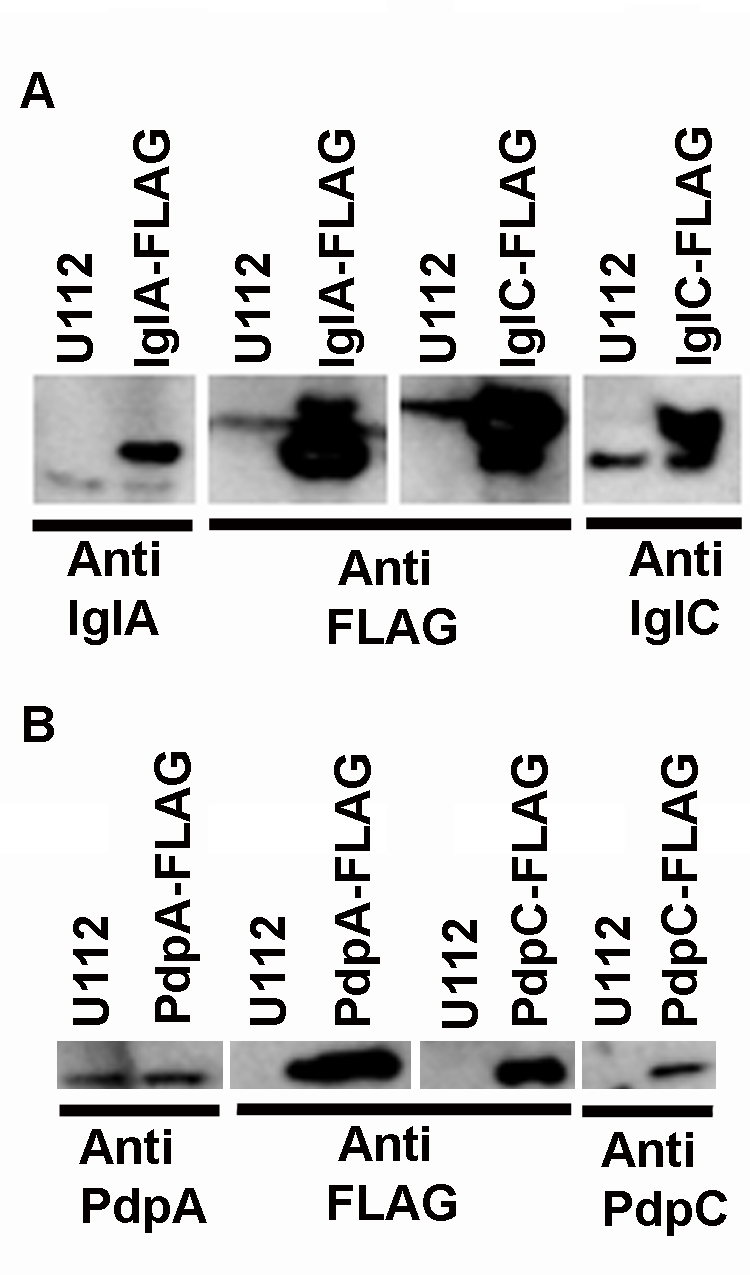

Supplement: Figure S2 — Native and FLAG-tagged FPI Proteins. Western blot of F. novicida U112 wild type and U112 expressing the respective C-terminally tagged FPI proteins from the Francisella expression plasmid are labeled above each lane. (A) 15% gel with IglAC, (B) 8% gel with PdpAC. IglAC and PdpAC proteins were detected with polyclonal rabbit anti IglAC and PdpAC. FLAG-tagged proteins are detected with monoclonal mouse anti FLAG, goat anti mouse conjugated HRP, and chemiluminescent substrate. (TIF) [file pone.0105773.s002.tif]

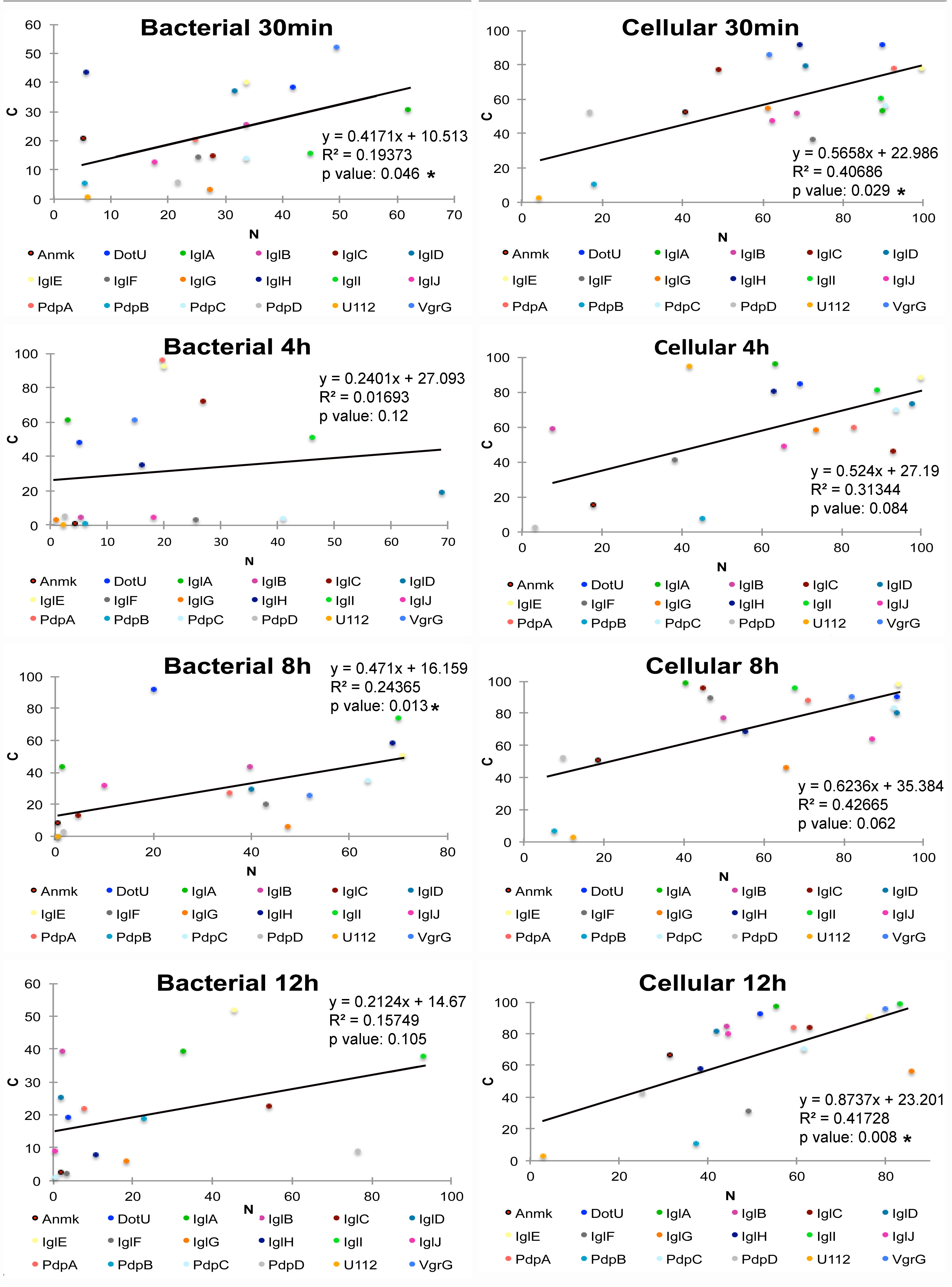

Supplement: Figure S4 — N- and C-tag Correlation. The data for N- and C-tagged proteins in both bacterial and cellular analyses were plotted against each other for each protein, at each time point. The data were subjected to a Spearman correlation tests. A best-fit trend line was inserted along with the slope, R2 values, and the P value. Asterisks indicate significance (p≤0.05) of the Spearman's test for correlation. Specific analysis and times points are indicated on graphs. (TIF) [file pone.0105773.s004.tif]
