# Supplementary material for: Francisella novicida Pathogenicity Island Encoded Proteins Were Secreted during Infection of Macrophage-Like Cells
Source: PLoS One. 2014 Aug 26;9(8):e105773. doi: 10.1371/journal.pone.0105773 (PMC4144950; doi:10.1371/journal.pone.0105773)
Supplement: Table S1 — Molecular weights of FPI proteins. The molecular weights of F. novicida FPI encoded proteins. (DOCX) [file pone.0105773.s005.docx]

**S. Table 1: The molecular weights of *F. novicida* FPI encoded proteins are listed in this table.**

| FPI Protein | Molecular weight kDa |
| --- | --- |
| PdpA | 95 |
| PdpB | 127.5 |
| IglE | 14.5 |
| VgrG | 17.5 |
| IglF | 67.6 |
| IglG | 18.4 |
| IglH | 55.3 |
| DotU | 24.6 |
| IglI | 44.6 |
| IglJ | 30.9 |
| PdpC | 156 |
| PdpE | 22 |
| IglD | 46.5 |
| IglC | 22.4 |
| IglB | 58.9 |
| IglA | 21 |
| PdpD | 135.4 |
| Anmk | 40.5 |
