# Supplementary material for: Francisella novicida Pathogenicity Island Encoded Proteins Were Secreted during Infection of Macrophage-Like Cells
Source: PLoS One. 2014 Aug 26;9(8):e105773. doi: 10.1371/journal.pone.0105773 (PMC4144950; doi:10.1371/journal.pone.0105773)
Supplement: Table S2 — Means of FLAG with bacteria and FLAG within cells and significance. Each of the FPI proteins were examined for their localization with bacteria or within infected host cells, which is indicated as Bacterial or Cellular in the analysis column. Within each analysis proteins were examined via the FLAG tag on the N-terminus and the C-terminus. Values indicate the mean percentage of bacterial-FLAG co-localization or the mean percentage of infected cells containing FLAG from 3 independent experiments. Significance was determined with a left sided Dunett's test, * p<0.05, **p<0.001, and ***p<0.0001. (DOCX) [file pone.0105773.s006.docx]

**Table S2: Means of FLAG with bacteria and FLAG within cells and significance.**

| **Protein** | **Analysis** | **Tag** | **30min** | **4h** | **8h** | **12h** |
| --- | --- | --- | --- | --- | --- | --- |
| PdpA | Cellular | N | 92.56* | 82.90* | 71.10 | 59.13 |
|  |  | C | 77.64*** | 60.08*** | 88.19*** | 83.96* |
|  | Bacterial | N | 24.68 | 19.60 | 35.40 | 7.85 |
|  |  | C | 20.62 | 96.27* | 27.49 | 22.02 |
| PdpB | Cellular | N | 17.90 | 45.06 | 7.576 | 37.20 |
|  |  | C | 10.28 | 7.90 | 6.81 | 11.11 |
|  | Bacterial | N | 5.50 | 6.14 | 0.50 | 4.01 |
|  |  | C | 5.39 | 1.22 | 0.12 | ND |
| PdpC | Cellular | N | 90.54* | 93.75* | 92.59 | 61.49 |
|  |  | C | 56.00* | 69.83 | 83.44*** | 70.83* |
|  | Bacterial | N | 33.55* | 40.95* | 63.80 | 22.8* |
|  |  | C | 14.22 | 3.72 | 34.70 | 18.94 |
| PdpD | Cellular | N | 16.97 | ND | 9.58 | 25.05 |
|  |  | C | 52.78* | 25.00 | 52.82 | 42.95 |
|  | Bacterial | N | 21.65 | 2.40 | 1.80 | 0.32 |
|  |  | C | 5.78 | 5.41 | 2.66 | 0.98 |
| PdpE | Cellular | N | 33.70 | 8.33 | 2.38 | 25.96 |
|  |  | C | 86.11*** | 69.99 | 97.36*** | 99.78** |
|  | Bacterial | N | 4.75 | 30.00 | 30.15 | 10.75 |
|  |  | C | 27.50* | 4.70 | 37.55 | 36.57* |
| IglA | Cellular | N | 89.68* | 63.33 | 40.51 | 55.37 |
|  |  | C | 53.63* | 96.08* | 98.68*** | 97.53*** |
|  | Bacterial | N | 61.97*** | 3.08 | 1.32 | 32.60** |
|  |  | C | 30.74** | 61.59* | 43.47* | 39.32** |
| IglB | Cellular | N | 68.55 | 7.39 | 49.69 | 44.30 |
|  |  | C | 52.06* | 58.77 | 77.14** | 84.72* |
|  | Bacterial | N | 33.65* | 5.40 | 39.70 | 2.41 |
|  |  | C | 25.49** | 4.62 | 43.46* | 39.32** |
| IglC | Cellular | N | 48.83 | 92.86* | 44.88 | 62.97 |
|  |  | C | 77.15*** | 46.03 | 95.59*** | 84.09* |
|  | Bacterial | N | 27.75 | 26.80 | 4.55 | 54.30*** |
|  |  | C | 14.88 | 71.95 | 12.73 | 22.76 |
| IglD | Cellular | N | 70.66 | 97.73* | 93.14 | 41.93 |
|  |  | C | 79.66 | 73.59 | 80.03*** | 81.49* |
|  | Bacterial | N | 31.67 | 68.80*** | 40.10 | 2.00 |
|  |  | C | 37.28** | 19.38 | 29.78 | 25.32 |
| IglE | Cellular | N | 99.47** | 99.70* | 93.59 | 76.26 |
|  |  | C | 77.82*** | 88.58* | 97.83*** | 91.30* |
|  | Bacterial | N | 33.59* | 20.00 | 70.75* | 45.30*** |
|  |  | C | 40.28*** | 92.34 | 50.65* | 51.84*** |
| IglF | Cellular | N | 72.29 | 38.33 | 46.60 | 49.07 |
|  |  | C | 36.57 | 41.23 | 89.44 | 31.11 |
|  | Bacterial | N | 25.28 | 25.51 | 42.95 | 3.32 |
|  |  | C | 14.57 | 3.61 | 19.77 | 2.20 |
| IglG | Cellular | N | 61.11 | 73.41 | 65.39 | 85.92 |
|  |  | C | 54.62 | 58.00 | 46.59 | 56.51 |
|  | Bacterial | N | 27.20 | 1.05 | 47.35 | 18.45 |
|  |  | C | 3.24 | 3.61 | 6.02 | 6.04 |
| IglH | Cellular | N | 69.16 | 62.96 | 55.37 | 38.40 |
|  |  | C | 91.92*** | 80.73* | 68.61* | 58.32 |
|  | Bacterial | N | 5.70 | 16.12 | 68.90* | 10.75 |
|  |  | C | 43.63*** | 35.48 | 58.03** | 8.02 |
| IglI | Cellular | N | 89.38* | 88.89 | 67.71 | 83.24 |
|  |  | C | 60.88 | 81.00* | 95.45*** | 99.07** |
|  | Bacterial | N | 44.76*** | 45.97** | 69.90* | 93.0*** |
|  |  | C | 15.84 | 50.91* | 73.59** | 37.85** |
| IglJ | Cellular | N | 62.44 | 65.52 | 87.05 | 44.59 |
|  |  | C | 47.58 | 49.36 | 64.38* | 80.16* |
|  | Bacterial | N | 17.57 | 18.15 | 9.90 | 0.30 |
|  |  | C | 12.66 | 4.60 | 31.84 | 9.04 |
| VgrG | Cellular | N | 61.55 | 41.67 | 81.85 | 80.00 |
|  |  | C | 86.05*** | 94.87* | 90.72*** | 95.74* |
|  | Bacterial | N | 49.47*** | 14.872 | 51.97 | 76.60*** |
|  |  | C | 51.92*** | 61.22*** | 25.22 | 9.04 |
| DotU | Cellular | N | 89.69* | 69.51 | 93.33 | 51.77 |
|  |  | C | 91.92** | 84.95* | 90.21*** | 92.27* |
|  | Bacterial | N | 41.69** | 5.00 | 20.15 | 3.80 |
|  |  | C | 38.25** | 48.57* | 91.65*** | 19.20 |
| Anmk | Cellular | N | 40.73 | 17.89 | 18.52 | 31.47 |
|  |  | C | 52.57* | 15.36 | 50.78 | 67.00* |
|  | Bacterial | N | 5.2 | 4.25 | 0.45 | 2.08 |
|  |  | C | 20.99 | 1.12 | 8.05 | 2.69 |
| U112 | Cellular | N | 4.28 | 3.12 | 12.17 | 2.96 |
|  |  | C | 2.94 | 2.97 | 2.94 | 2.98 |
|  | Bacterial | N | 5.85 | 2.33 | 0.64 | ND |
|  |  | C | 0.65 | 0.43 | 0.19 | 1.02 |
